# Turning Waste into Treasure: Functionalized Biomass‐Derived Carbon Dots for Superselective Visualization and Eradication of Gram‐Positive Bacteria

**DOI:** 10.1002/advs.202411084

**Published:** 2025-01-24

**Authors:** Ke‐Fei Xu, Zihao Wang, Macheng Cui, Yuhan Jiang, Chengcheng Li, Zi‐Xi Wang, Ling‐Yi Li, Chenyang Jia, Lijie Zhang, Fu‐Gen Wu

**Affiliations:** ^1^ State Key Laboratory of Digital Medical Engineering Jiangsu Key Laboratory for Biomaterials and Devices School of Biological Science and Medical Engineering Southeast University 2 Southeast University Road Nanjing 211189 P. R. China; ^2^ Mudi Meng Honors College China Pharmaceutical University Longmian Dadao Road Nanjing 211189 P. R. China; ^3^ International Innovation Center for Forest Chemicals and Materials and Jiangsu Co‐Innovation Center for Efficient Processing and Utilization of Forest Resources Nanjing Forestry University Nanjing 210037 P. R. China; ^4^ Department of Urology Zhongda Hospital Southeast University Nanjing Jiangsu 210009 P. R. China; ^5^ Department of Obstetrics and Gynecology Zhongda Hospital Southeast University 87 Dingjiaqiao Nanjing 210009 P. R. China

**Keywords:** antibacterial therapy, bacterial imaging, biomass carbon dots, cell wall‐targeting probes, wound healing

## Abstract

Gram‐positive bacteria pose significant threats to human health, necessitating the development of targeted bacterial detection and eradication strategies. Nevertheless, current approaches often suffer from poor targeting specificity. Herein, the study utilizes purple rice lixivium to synthesize biomass carbon dots (termed BCDs) with wheat germ agglutinin‐like residues for precisely targeting Gram‐positive bacteria. Subsequently, fluorescein isothiocyanate (FITC) molecules are grafted onto BCDs to yield FITC‐labeled BCDs (termed CDFs), which can selectively and rapidly (≤5 min) stain bacterial cell wall and particularly target the peptidoglycan component. Strikingly, CDFs achieve superselective visualization of Gram‐positive bacteria even in the presence of mammalian cells and Gram‐negative bacteria. Furthermore, protoporphyrin (PpIX) molecules are conjugated onto BCDs to yield PpIX‐modified BCDs (termed CDPs), which can induce bacterial aggregation and in situ generate singlet oxygen for realizing enhanced antibacterial photodynamic therapy (PDT). At the minimum bactericidal concentration of CDPs (PpIX: 5 µg mL^−1^), CDP‐mediated PDT disrupts bacterial structure and metabolism pathways, thereby affecting bacterial interactions to eradicate biofilms. Importantly, CDP‐mediated PDT efficiently modulates antiinflammatory responses to promote wound healing in the bacteria‐infected mice. This study underscores the significance of harnessing renewable and cost‐effective biomass resources for preparing Gram‐positive bacteria‐targeting theranostic agents, which may find potential clinical applications in the future.

## Graphical abstract

This research develops a type of purple rice lixivium‐derived biomass carbon dots (BCDs), which present excellent affinity to Gram‐positive bacteria due to the presence of WGA‐like residues. Inspired by this finding, this research further fabricates FITC‐labeled and PpIX‐modified BCDs to achieve superselective visualization and eradication of Gram‐positive bacteria even in the presence of mammalian cells and Gram‐negative bacteria.

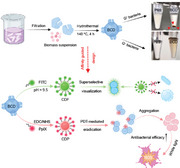

## 1 Introduction

Since the establishment of the Gram staining method in 1884, bacteria have been classified into two main types: Gram‐positive (G^+^) and Gram‐negative (G^−^) ones, primarily distinguished by their surface components.^[^ ^1^ ^]^ Specifically, the Gram staining process involves the fixing, staining, and washing steps. However, these steps are complicated and potentially leads to cell loss and false positive results.^[^ ^2^ ^]^ Recently, the fluorescence imaging technology has attracted significant attention in the biomedical field due to its operational simplicity, high sensitivity, and capability to monitor complex biological processes in real time.^[^ ^3^ ^]^ Consequently, various fluorescent probes have been developed for detecting bacteria to overcome the limitations of the Gram staining method. Typically, the design of bacterial staining probes focuses on their capacity to target specific bacterial components. These components include 1) the negatively charged bacterial cell wall,^[^ ^4^ ^]^ 2) bacterial proteins,^[^ ^5^ ^]^ 3) bacterial nucleic acids,^[^ ^6^ ^]^ and 4) specific enzymes.^[^ ^7^ ^]^ Notably, G^+^ bacteria are the pathogens that significantly impact millions of lives worldwide, particularly in developing countries.^[^ ^8^ ^]^ Therefore, cost‐effective, precise, and rapid detection of G^+^ bacteria is crucial in clinical settings. To distinguish G^+^ bacteria from G^−^ bacteria, researchers have rationally designed fluorescent probes that are capable of binding to the specific components of G^+^ bacterial cell walls.^[^ ^9^ ^]^ In detail, the cell wall of G^+^ bacteria predominantly consists of thick peptidoglycan complexes, providing structural stability and protection. In contrast, G^−^ bacteria possess a thinner peptidoglycan layer beneath an outer membrane containing lipopolysaccharide.^[^ ^10^ ^]^ Accordingly, several probes have been developed to target the peptidoglycan layer, facilitating the selective imaging of G^+^ bacteria. For example, Sizemore et al. employed fluorescein isothiocyanate (FITC)‐conjugated wheat germ agglutinin (WGA) to selectively stain G^+^ bacteria,^[^ ^11^ ^]^ as WGA can specifically bind to the *N*‐acetylglucosamine within the peptidoglycan layer. However, WGA‐based fluorescent probes require washing steps to enhance the signal‐to‐noise ratio and a high concentration of potassium chloride to augment fluorescence intensity.^[^ ^12^ ^]^ Moreover, a recent study has demonstrated that some WGA‐based probes also stain G^−^ bacteria,^[^ ^13^ ^]^ indicating the limited specificity of WGA for targeting G^+^ bacteria. Although several other fluorescent probes have also been developed for selective imaging of G^+^ bacteria,^[^ ^14^ ^]^ many of them are expensive and exhibit undesired targeting specificity. Hence, there is an urgent demand for inexpensive fluorescent probes capable of rapidly and specifically labeling G^+^ bacteria. Ultimately, these novel probes can facilitate effective medical treatment and reduce healthcare costs.

Carbon dots (CDs) represent a novel class of 0D materials that are garnering growing interest in the biomedical field. Importantly, CDs have been widely used in bioimaging due to their remarkable photostability, good biocompatibility, and adjustable fluorescence properties.^[^ ^15^ ^]^ Biomass carbon dots (BCDs) refer to a subset of CDs derived from renewable biomass sources, such as plant‐ and animal‐derived bioproducts, using straightforward and environmentally friendly synthesis methods.^[^ ^16^ ^]^ In comparison with other carbon sources, biomass carbon sources are eco‐friendly natural products. These products offer several advantages in the preparation of BCDs, including cost‐effectiveness, widespread availability, and environmental sustainability.^[^ ^17^ ^]^ Additionally, the fabrication of BCDs facilitates the conversion of low‐value biomass waste into valuable materials, thereby generating economic benefits through waste utilization.^[^ ^18^ ^]^ Over recent years, diverse biomass sources, such as rice husks,^[^ ^19^ ^]^ winter melon,^[^ ^20^ ^]^ silkworm chrysalis,^[^ ^21^ ^]^ baked lamb,^[^ ^22^ ^]^ goose feather,^[^ ^23^ ^]^ and lychee seeds,^[^ ^24^ ^]^ have been employed to prepare BCDs. Typically, BCDs possess abundant chemical groups on their surfaces, enabling facile surface modification with functional molecules.^[^ ^25^ ^]^ By grafting fluorescent moieties onto the surfaces of BCDs, the fluorescence properties of BCDs can be finely adjusted for bioimaging.^[^ ^26^ ^]^ Similarly, conjugating therapeutic agents with BCDs facilitates drug delivery and disease treatment.^[^ ^27^ ^]^ Specifically, BCDs have been widely used for antibacterial treatment.^[^ ^28^ ^]^ For example, garlic was utilized to prepare antimicrobial CDs.^[^ ^29^ ^]^ These outstanding characteristics endow BCDs with broad application potential in the biomedical field.

**Figure 1:**
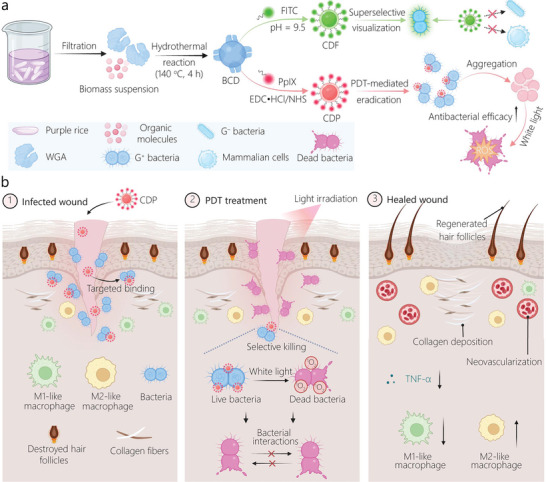
Schematic illustrating the synthesis of functionalized BCDs (i.e., CDFs and CDPs) and their applications for G^+^ bacteria‐targeted imaging/eradication and wound healing promotion. a) Scheme showing the preparation of CDFs and CDPs and their respective applications for selective imaging and eradication of G^+^ bacteria. b) Scheme depicting the CDP‐mediated PDT for modulating antiinflammatory responses and promoting the wound healing process of bacteria‐infected mice.

In this study, we utilize the purple rice lixivium to fabricate a novel type of blue‐emissive BCDs via a one‐step hydrothermal reaction (**Figure** **1**a). Remarkably, BCDs possess superselective binding affinity toward G^+^ bacteria, potentially attributed to the presence of WGA‐like residues and functional groups (e.g., amino groups) on their surfaces. Specifically, the WGA‐like residues can specifically target the *N*‐acetylglucosamine within the bacterial surface,^[^ ^30^ ^]^ and the amino groups contribute to hydrogen bonding interactions with bacterial surfaces.^[^ ^31^ ^]^ To improve the fluorescence property of BCDs, we proceed to conjugate FITC molecules with the amino groups present on the surfaces of the blue‐emissive BCDs to obtain FITC‐labeled BCDs (termed CDFs). Compared with BCDs, CDFs exhibit bright green fluorescence, effectively minimizing interference from biological autofluorescence and elevating their suitability for bioimaging. Notably, the CDF nanoprobe primarily binds to the peptidoglycan component of the bacterial cell wall, facilitating the selective visualization of G^+^ bacteria. Furthermore, CDFs exhibit significant G^+^ bacteria‐targeting specificity in the presence of mammalian cells or G^−^ bacteria, thereby enabling the rapid and precise detection of bacterial infection (caused by G^+^ bacteria) in blood samples. Inspired by the advantageous G^+^ bacteria‐targeting specificity of BCDs, we further develop a facile antibacterial strategy. Specifically, we modify BCDs with the photosensitizer protoporphyrin (PpIX) to yield PpIX‐conjugated BCDs (termed CDPs) for antibacterial photodynamic therapy (PDT). As expected, CDPs efficiently bind to the cellular surface of Gram‐positive *Staphylococcus aureus* (*S*. *aureus*), inducing bacterial aggregation and subsequently enhancing bacterial eradication through PDT‐mediated reactive oxygen species (ROS) generation. Remarkably, the ROS generated in situ disrupt bacterial cells and influence intracellular metabolism to alter bacterial interactions and achieve biofilm eradication, which can be demonstrated by crystal violet and live/dead staining methods.^[^ ^32^ ^]^ Consequently, CDP‐mediated PDT successfully eliminates bacteria and promotes wound healing in a bacteria‐infected mouse model (Figure 1b). Given the facile and cost‐effective synthesis of functional BCD‐based nanoplatforms (i.e., CDF and CDP), we believe that our strategies of CDF‐ and CDP‐based G^+^ bacteria‐targeted imaging and eradication may hold great potential for practical applications.

## 2 Results and Discussion

### 2.1 Characterization of BCDs

**Figure 2:**
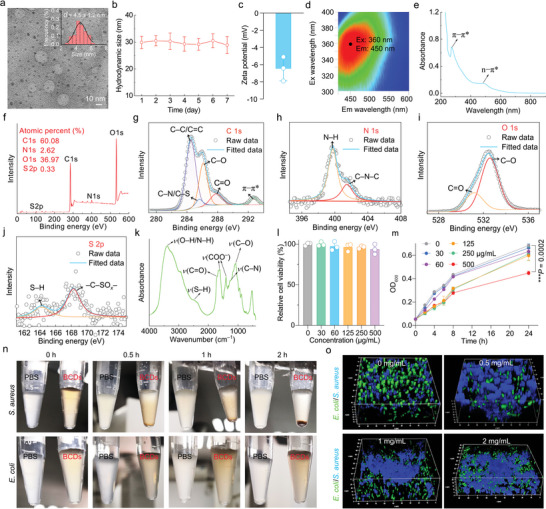
Characterization of BCDs. a) TEM image and corresponding size distribution histogram of BCDs. b) Hydrodynamic diameters of BCDs (dispersed in water) within one week. c) Zeta potential result of BCDs dispersed in PBS (10 mM, pH = 7.4). Data in (b) and (c) are presented as mean ± standard deviation (SD), *n * =  3 experimental replicates. d) Excitation–emission contour plot of BCDs (dispersed in PBS). e) UV–vis absorption spectrum of BCDs (dispersed in PBS). f) Survey XPS curve of BCDs and the high‐resolution XPS curves of g) C 1s, h) N 1s, i) O 1s, and j) S 2p. k) FTIR spectrum of BCDs. l) Relative viabilities of 4T1 cells incubated with different concentrations of BCDs for 24 h. m) Growth curves of *S*. *aureus* incubated with different concentrations of BCDs for 24 h. Data in (l) and (m) are presented as mean ± SD. *n* = 3 experimental repeats. n) Photographs of centrifuge tubes containing *S*. *aureus* or *E*. *coli* (OD_600_ = 1) suspensions in the absence and presence of BCDs (0.5 mg mL^−1^) for different time periods. o) 3D confocal fluorescence images of mixed bacteria (*S*. *aureus* and *E*. *coli*) incubated with different concentrations of BCDs (0, 0.5, 1, and 2 mg mL^−1^) for 2 h. *S*. *aureus* and *E*. *coli* were labeled by Hoechst 33342 (blue fluorescence) and SYTO 9 (green fluorescence), respectively.

As depicted in Figure 1a, BCDs were synthesized using purple rice lixivium via a one‐step hydrothermal method (140 °C, 4 h). Transmission electron microscopy (TEM) imaging revealed that BCDs possess an average size of 4.5 ± 1.2 nm (**Figure** **2**a). Dynamic light scattering results confirmed the excellent dispersibility and stability of BCDs in water for at least one week (Figure 2b), with the zeta potential measured to be –6.4 ± 1.3 mV (Figure 2c). Notably, BCDs displayed an optimal excitation wavelength at 360 nm, accompanied by a corresponding blue fluorescence emission wavelength at 450 nm (Figure 2d). According to the ultraviolet−visible (UV−vis) absorption spectrum, BCDs exhibited an absorption peak at ≈270 nm (Figure 2e), attributed to the π−π* transition of the aromatic C═C bond. The broad absorption band in the 400−550 nm range can be attributed to the n−π* transition involving the C═O and C−N bonds.^[^ ^33^ ^]^ Besides, X‐ray photoelectron spectroscopy (XPS) and Fourier transform infrared (FTIR) spectroscopy were employed to investigate the chemical composition of BCDs. The full XPS curve revealed the presence of carbon (C), nitrogen (N), oxygen (O), and sulfur (S), with the atomic percentages of 60.08%, 2.62%, 36.97%, and 0.33%, respectively (Figure 2f). The high‐resolution C 1s spectrum displays peaks corresponding to C═C/C−C (284.5 eV), C−N/C−S (285.6 eV), C−O (286.1 eV), C═O (287.8 eV), and a π−π* shake‐up peak (292.7 eV) (Figure 2g). The N 1s band can be deconvoluted into two peaks at 399.7 and 401.5 eV, representing N−H and C−N−C, respectively (Figure 2h). The O 1s spectrum contains two peaks at 531.2 and 532.6 eV from C═O and C−O, respectively (Figure 2i). The S 2p bands can be divided into two peaks at 164.2 and 168.3 eV, representing the S−H and −C−SO*_x_*− groups, respectively (Figure 2j). In the FTIR spectrum (Figure 2k), the stretching vibrations of N−H and O−H bonds were observed at ∼3400 cm^−1^. Additionally, the peaks at 1075, 1258, 1402/1618, and 1661/1709 cm^−1^ indicated the stretching vibrations of C−O, C−N, COO^−^, and C═O, respectively. Besides, the band located in 2600−2500 cm^−1^ can be ascribed to the stretching vibration of S−H. The results of 3‐(4,5‐dimethyl‐2‐thiazolyl)‐2,5‐diphenyl‐2*H*‐tetrazolium bromide (MTT) assay suggested the good cytocompatibility of BCDs toward 4T1 cells (a murine mammary carcinoma cell line) (Figure 2l). Besides, BCDs had a negligible hemolytic effect on red blood cells (RBCs) (Figure S1, Supporting Information), indicating its good hemocompatibility. To evaluate the potential toxicity of BCDs toward bacteria, different concentrations of BCDs were incubated with Gram‐positive *S*. *aureus* and Gram‐negative *Escherichia coli* (*E*. *coli*), respectively. Intriguingly, 0.5 mg/mL of BCDs presented negligible impact on the viability of *E*. *coli* (Figure S2, Supporting Information), while the same concentration of BCDs significantly suppressed the proliferation of *S*. *aureus* (Figure 2m). Taken together, BCDs demonstrated favorable biocompatibility toward 4T1 cells (mammalian cells) and *E*. *coli* cells (G^−^ bacteria) but exhibited slight toxicity toward *S. aureus* cells (G^+^ bacteria). Therefore, it is postulated that BCDs may selectively interact with the specific structures of G^+^ bacteria, thereby inhibiting the activities of *S. aureus*.

To further study the potential interactions between bacteria and BCDs, we separately incubated *S*. *aureus* and *E*. *coli* with BCDs. As the incubation time increased, evident aggregation of *S*. *aureus* cells was only observed in the BCDs‐treated group, as evidenced by pronounced sedimentation at the bottom of the centrifuge tube (Figure 2n). In contrast, *E*. *coli* in both phosphate‐buffered saline (PBS) and BCD suspension remained well dispersed throughout the observation period (Figure 2n). Confocal imaging results corroborated the aggregation of *S*. *aureus* in the BCD suspension (Figure S3, Supporting Information). To assess the bacterial binding selectivity of BCDs, we exposed them to a suspension containing both *S*. *aureus* and *E*. *coli*. With prolonged incubation, *S*. *aureus* gradually aggregated and eventually formed massive bacterial aggregates (Figure S4, Supporting Information), a phenomenon absent in the control group (Figure S5, Supporting Information). To maintain the integrity of bacterial aggregates and capture the native states of the treated bacteria, we employed 3D imaging technology for direct visualization of bacterial suspensions. In the control group (BCDs: 0 mg/mL), *S*. *aureus* and *E*. *coli* were distinctly dispersed in the PBS (Figure 2o). Nevertheless, with the increase of the BCD concentration, an impressive phenomenon occurred: *S*. *aureus* exhibited pronounced aggregation, while *E*. *coli* remained uniformly dispersed in the solution (Figure 2o), implying the potential affinity of BCDs to *S*. *aureus*. Interestingly, BCDs also induced the aggregation of *S*. *aureus* in a mixed suspension of *S*. *aureus* and mammalian RBCs (Figure S6, Supporting Information), thus further emphasizing the potential specificity of BCDs toward G^+^ bacteria. Collectively, these results demonstrated the exceptional superselective binding capacity of BCDs toward G^+^ bacteria among *S*. *aureus*, *E*. *coli*, and RBCs.

According to the reported studies,^[^ ^12^, ^13^ ^]^ WGA can specifically bind to the *N*‐acetylglucosamine residues present on the surfaces of bacterial cells. We hypothesized that the surface of BCDs may be enriched with similar functional structures to WGA, which may be derived from the purple rice lixivium. Consequently, we quantified the WGA content in both the lixivium and BCDs using an enzyme‐linked immunosorbent assay (ELISA) kit. According to the standard curve (Figure S7, Supporting Information), the concentrations of WGA residues in the lixivium (100 µg mL^−1^) and BCDs (0.5 mg mL^−1^) were determined to be 5.16 and 4.08 ng mL^−1^, respectively. Hence, the presence of WGA residues likely contributes to the G^+^ bacteria‐targeting ability of BCDs. Notably, the purple rice lixivium failed to induce aggregation or inhibit the growth of *S*. *aureus* (Figure S8, Supporting Information), thereby illustrating the superior affinity of BCDs to *S*. *aureus*. Taken together, BCDs exhibited remarkable specificity toward G^+^ bacteria, overcoming the potential limitations of WGA molecules, which may also bind to G^−^ bacteria.^[^ ^13^ ^]^

Subsequently, we verified the presence of abundant amino groups within BCDs through the fluorescamine‐based detection method. In detail, fluorescamine reacts with substances/molecules possessing primary amino groups to generate fluorescence signal. Meanwhile, the intensity of the fluorescence signal is proportional to the concentration of primary amino groups. Herein, glycine was chosen as the reference, and the fluorescence intensities of fluorescamine‐treated glycine solutions presented desirable linearity with the concentrations of glycine (Figure S9a,b, Supporting Information). Besides, the presence of amino groups within BCDs was confirmed by the fluorescence spectrum of fluorescamine‐treated BCDs (Figure S9c, Supporting Information). To study the potential hydrogen bonding between amino groups and bacterial surface, we used urea to disrupt the potential hydrogen bonding between BCDs and *S*. *aureus*. If the hydrogen bonding between BCDs and bacteria is disrupted by urea, fewer bacteria are expected to aggregate, resulting in a higher bacterial count in the suspension. Consistent with this hypothesis, the “BCDs + urea” group exhibited significantly more bacteria in the suspension compared to the “BCDs” group (Figure S9d, Supporting Information), suggesting reduced binding affinity of BCDs toward *S*. *aureus* due to urea treatment. These results suggest that functional groups, such as amino groups, contribute to the binding interaction of BCDs with bacterial cells. Collectively, BCDs presented a high affinity for G^+^ bacteria, while the presence of surface amino groups can facilitate their functionalization for further biomedical applications.

### 2.2 CDFs for Superselective Imaging of G^+^ Bacteria

**Figure 3:**
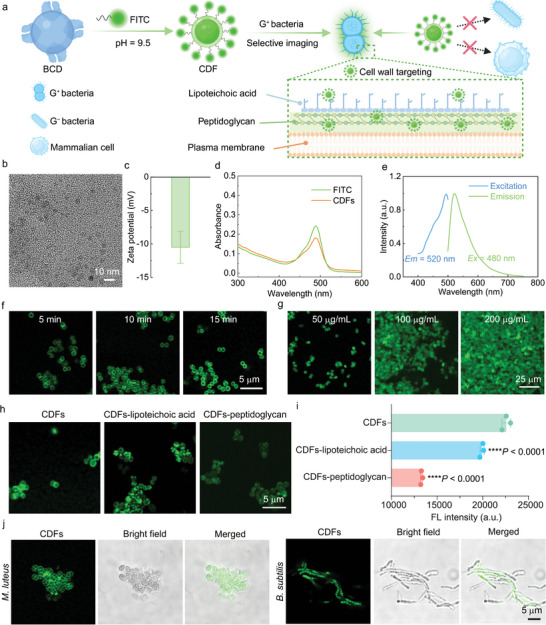
Characterization of CDFs. a) Schematic illustrating the fabrication of the CDF nanoprobe and its application for selective imaging of G^+^ bacteria. b) TEM image of CDFs. c) Zeta potential of CDFs dispersed in PBS (10 mM, pH = 7.4). d) UV–vis absorption spectra of FITC (dispersed in dimethyl sulfoxide (DMSO)) and CDFs (dispersed in a mixture of DMSO and water (19 : 1, vol/vol)). e) Excitation and emission fluorescence spectra of CDFs (dispersed in a mixture of DMSO and water (19 : 1, vol/vol)) collected at the corresponding optimal emission and excitation wavelengths. f) Confocal images of *S*. *aureus* stained by 50 µg mL^−1^ CDFs for different time periods. g) Confocal images of *S*. *aureus* stained by different concentrations of CDFs for 5 min. h) Confocal images of *S*. *aureus* stained by different CDF nanoprobes. CDFs: CDFs without any pretreatment. CDFs‐lipoteichoic acid: CDFs preincubated with 1 mg/mL lipoteichoic acid for 1 h. CDFs‐peptidoglycan: CDFs preincubated with 1 mg mL^−1^ peptidoglycan for 1 h. i) Corresponding flow cytometry analysis results of the bacterial cells treated similarly as the cells in (h). Data are presented as mean ± SD. *n* = 3 experimental repeats. Statistical significance in (i) was calculated via one‐way analysis of variance (ANOVA) with a Tukey's post‐hoc test. ^****^ *p* < 0.0001. j) Confocal images of different G^+^ bacteria (*M*. *luteus* and *B*. *subtilis*) stained by 50 µg mL^−1^ CDFs for 5 min.

Encouraged by the superior G^+^ bacteria‐targeting capacity of BCDs, we grafted FITC molecules onto BCDs to generate FITC‐conjugated BCDs (termed CDFs) for the superselective imaging of G^+^ bacteria (**Figure** **3**a). Owing to the small molecular weight of FITC, the size and morphology of CDFs remained similar to those of BCDs (Figure 3b). Notably, CDFs displayed a more negatively charged surface compared with BCDs (Figures 2c and 3c) and exhibited an absorption peak (∼490 nm) similar to that of FITC (Figure 3d), indicating the successful FITC modification. Additionally, the optimal excitation and emission wavelengths of CDFs were identified to be 480 and 520 nm (Figure 3e), suggesting the green fluorescence emission of CDFs. Subsequently, we explored the optimal conditions for using CDFs to stain G^+^ bacteria such as *S*. *aureus*. Specifically, CDFs efficiently labeled the bacterial surfaces of *S*. *aureus* within 5 min (Figure 3f; Figure S10, Supporting Information), with the suitable staining concentration determined to be 50 µg mL^−1^ (Figure 3g). Interestingly, we found that a high concentration of CDFs (200 µg mL^−1^) also resulted in significant aggregation of *S*. *aureus* cells (Figure 3g). Meanwhile, the surface of each individual bacterium remained effectively stained (Figure 3g), highlighting the exceptional potency of CDFs in labeling the bacterial cell wall. To confirm the CDF‐binding sites on the bacterial surfaces, we separately incubated two major components of G^+^ bacterial cell walls, namely lipoteichoic acid and peptidoglycan, with CDFs to block the potential bacteria‐targeting structures of CDFs. Afterward, *S*. *aureus* cells were stained by CDFs (set as the “CDFs” group), lipoteichoic acid‐treated CDFs (set as the “CDFs‐lipoteichoic acid” group), and peptidoglycan‐treated CDFs (set as the “CDFs‐peptidoglycan” group), respectively. Strikingly, the imaging quality of *S*. *aureus* in the “CDFs‐peptidoglycan” group significantly decreased compared with the other two groups (“CDFs” and “CDFs‐lipoteichoic acid”) (Figure 3h), which was also validated by the corresponding flow cytometric results (Figure 3i). This result indicated that the presence of WGA‐like residues endows BCDs with a strong affinity toward the *N*‐acetylglucosamine‐containing peptidoglycan layer. In contrast, the main components of lipoteichoic acid are phosphodiester‐linked glycerol phosphate and lipid, which cannot be recognized by WGA‐like residues. To evaluate the universal staining ability of CDFs toward G^+^ bacteria, we applied them to two other types of G^+^ bacteria (*Micrococcus luteus* (*M*. *luteus*) and *Bacillus subtilis* (*B*. *subtilis*) and three types of G^−^ bacteria (*E*. *coli*, *Pseudomonas aeruginosa* (*P*. *aeruginosa*), and *Proteus vulgaris* (*P*. *vulgaris*)). The imaging results revealed that CDFs exhibited desirable cell wall labeling performance toward G^+^ bacteria (Figure 3j), while they showed negligible interactions with G^−^ bacteria (Figure S11, Supporting Information).

**Figure 4:**
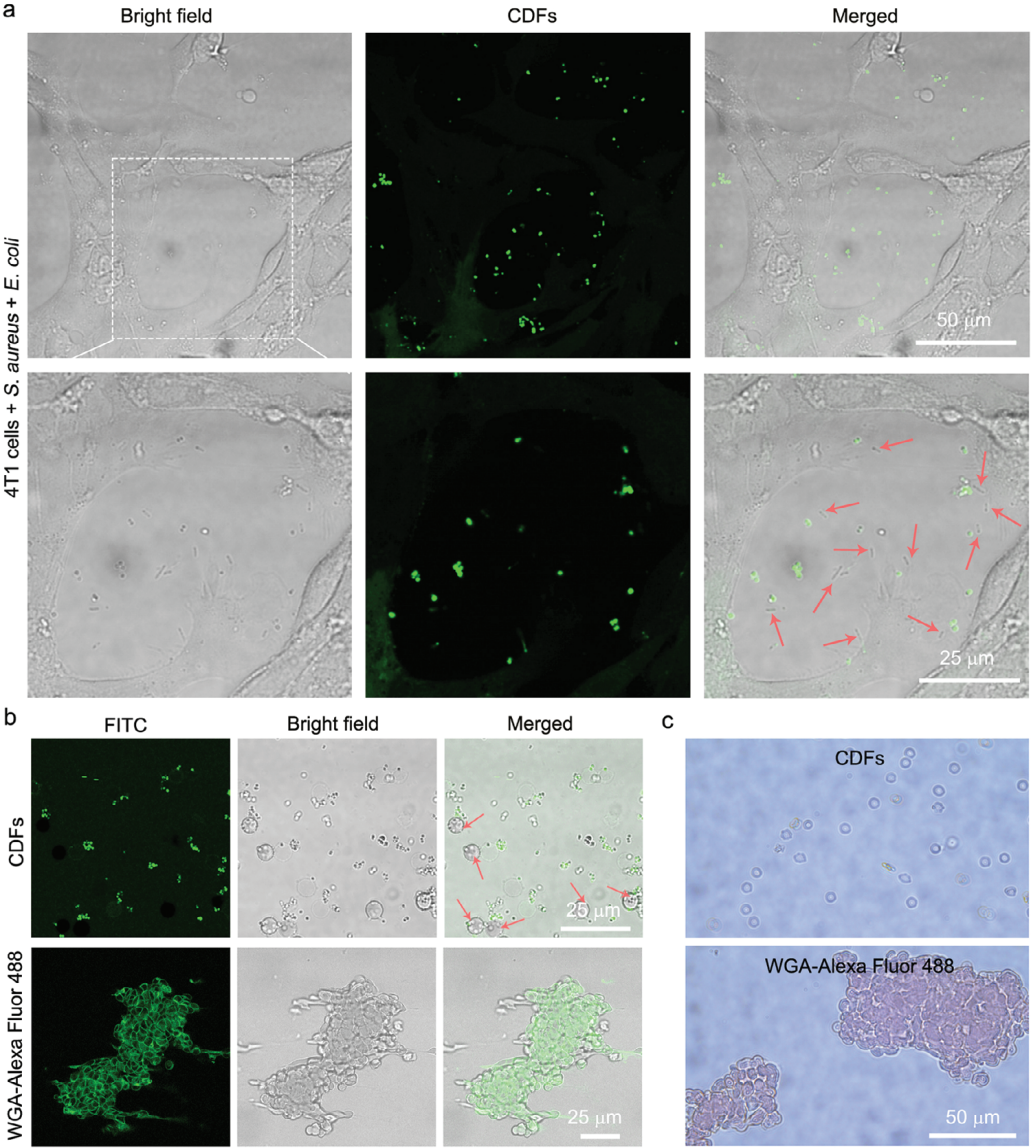
Analysis of CDF‐mediated G^+^ bacterium detection. a) Confocal images of mixed cells (4T1 cells, *E*. *coli*, and *S*. *aureus*) stained by 0.5 mg mL^−1^ CDFs for 15 min. The red arrows indicate the unstained *E*. *coli*. b) Confocal and c) optical images of the mixed cells (*S*. *aureus* and RBCs) stained by 0.5 mg mL^−1^ CDFs and 0.1 mg mL^−1^ WGA‐Alexa Fluor 488 for 15 min, respectively. The red arrows in (b) indicate the unstained RBCs.

To evaluate the G^+^ bacteria‐targeting specificity of CDFs, we incubated them with a mixture of mammalian cells (4T1 cells) and G^+^ bacteria (*S*. *aureus*). As expected, CDFs selectively labeled *S*. *aureus* with a high signal‐to‐background ratio (Figure S12a, Supporting Information). Subsequently, we conducted a comprehensive affinity assessment of CDFs when incubated with mammalian cells (4T1 cells), G^−^ bacteria (*E*. *coli*), and G^+^ bacteria (*S*. *aureus*). Impressively, as shown in **Figure** **4**a, only the *S*. *aureus* cells were notably stained by the CDFs, while the *E*. *coli* cells (as indicated by the arrows in the figure) and the 4T1 cells remained unstained. Motivated by the superior specificity of CDFs toward G^+^ bacteria, we applied them to detect *S*. *aureus* in RBC suspensions. As depicted in Figure S12b (Supporting Information), CDFs achieved rapid and accurate visualization of *S*. *aureus*, suggesting their potential for clinical detection of Gram‐positive bacterial infections in blood samples. By contrast, WGA‐Alexa Fluor 488 efficiently labeled the surfaces of RBCs (Figure 4b), probably due to the presence of *N*‐acetylglucosamine in the RBC plasma membrane. Besides, blood coagulation was observed in the WGA‐Alexa Fluor 488‐treated group (Figure 4c), indicating the limited application potential of fluorescent WGA conjugates in detecting bacterial infections. Different from WGA‐Alexa Fluor 488, CDFs only visualized and aggregated *S*. *aureus*, leaving RBCs unstained and uniformly dispersed (as marked by the arrows in Figure 4b). These results demonstrated the superior targeting specificity of CDFs toward *S. aureus* compared with the commercial dye (i.e., the fluorescent WGA conjugate). Collectively, the above results demonstrated that CDFs can serve as an excellent cell wall‐targeting fluorescent nanoprobe, holding great potential for superselective and rapid (≤5 min) imaging and detection of G^+^ bacteria.

### 2.3 CDP‐Mediated PDT for Antibacterial Treatment and Biofilm Eradication

Over the past decades, the overuse of antibiotics has resulted in a notable increase of drug‐resistant strains, posing a huge threat to human health.^[^ ^34^ ^]^ Consequently, there has been a burgeoning interest in exploring alternative antimicrobial strategies. Among these approaches, PDT has garnered attention due to its noninvasiveness and broad antibacterial spectrum.^[^ ^35^ ^]^ The PDT strategy typically involves the employment of photosensitizers, light irradiation, and oxygen to generate ROS.^[^ ^36^ ^]^ The ROS, generated during PDT, can disrupt essential bacterial structures, such as cell walls, leading to irreversible damage and eventual bacterial death.^[^ ^37^ ^]^ Importantly, bacteria exhibit limited resistance to ROS, rendering PDT a promising antibacterial strategy.^[^ ^38^ ^]^ Till date, PDT has found predominant applications in the clinical treatment of localized infectious diseases, such as oral bacterial infections.^[^ ^39^ ^]^ Benefiting from the CDs' advantages of easy surface modification, simple preparation, and good biocompatibility, the CD‐based PDT strategy has also been developed for combating various infectious bacteria.^[^ ^40^ ^]^ It is noteworthy that ROS may also elicit potential side effects in normal tissue cells, and therefore the development of therapeutic agents with superior bacteria‐targeting specificity is crucial for achieving effective and safe PDT‐mediated bacterial eradication in clinical settings.

**Figure 5:**
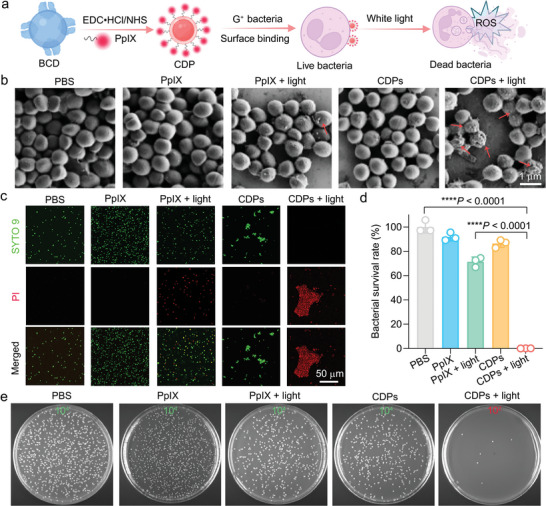
In vitro antibacterial effect of CDP‐mediated PDT. a) Schematic showing the synthesis of CDPs and CDP‐mediated antibacterial PDT. b) SEM images of *S*. *aureus* after different treatments. The red arrows indicate the dead *S*. *aureus* with broken cell walls. c) Confocal fluorescence images showing the *S*. *aureus* cells after different treatments as indicated. Before imaging, the bacteria in each group were stained by SYTO 9 (6 µm) and PI (30 µm) for 15 min. d) Survival rates of *S*. *aureus* after various treatments. Data are presented as mean ± SD. *n* = 3 experimental repeats. Statistical significance was calculated via one‐way ANOVA with a Tukey's post‐hoc test. ^****^ *p* < 0.0001. e) Representative photographs showing the agar plates of *S*. *aureus* after different treatments. The numbers (10^2^ and 10^4^) in the images indicate the dilution factors of the original bacterial suspensions. (b–e): White light (5 mW cm^−2^, 10 min) was used for PDT in the “PpIX + light” and “CDPs + light” groups.

Given the outstanding G^+^ bacteria‐targeting efficiency of BCDs or functionalized BCDs (i.e., CDFs), we grafted the photosensitizer PpIX onto BCDs to synthesize PpIX‐modified BCDs (termed CDPs) for PDT‐mediated antibacterial treatment (**Figure** **5**a). The PpIX content within CDPs was determined using the standard curve of free PpIX (Figure S13a, Supporting Information) and the UV–vis absorption spectrum of CDPs (Figure S13b, Supporting Information). Afterward, the levels of ROS (i.e., ^1^O_2_) generated by CDP‐ and PpIX‐mediated PDT were analyzed using the singlet oxygen sensor green (SOSG) reagent. The hydrophobic PpIX tends to aggregate in aqueous solutions, potentially limiting ROS generation during PDT. In contrast, PpIX molecules within CDPs are somewhat separately distributed on the surfaces of BCDs, leading to enhanced ROS generation (Figure S13c, Supporting Information). Moreover, CDPs presented a high affinity for bacterial surfaces, whereas free PpIX showed minimal interactions with the bacteria (Figure S14, Supporting Information). Strikingly, CDPs also induced the aggregation of *S*. *aureus*, similar to the effect observed with BCDs and CDFs. This aggregation phenomenon led to a higher local concentration of PpIX on/in the bacteria treated with CDPs compared with that on/in the bacteria treated with free PpIX, thereby amplifying ROS generation by CDP‐mediated PDT and ultimately resulting in enhanced antibacterial efficacy. Specifically, we visualized the ROS levels within bacteria using 2′,7′‐dichlorodihydrofluorescein diacetate (DCFH‐DA), and *S*. *aureus* in the “CDPs + light” group displayed the highest fluorescence intensity (Figure S15, Supporting Information), suggesting the abundant ROS generation induced by CDP‐mediated PDT. Encouraged by these findings, MIC_50_ (MIC: minimum inhibitory concentration) assessment was employed to investigate the lowest concentration of CDPs required to inhibit the growth of 50% bacteria. As shown in Figure S16 (Supporting Information), the MIC_50_ of PpIX in CDPs against *S*. *aureus* was found to be ∼5 µg/mL, whereas that of free PpIX against *S*. *aureus* exceeded 20 µg mL^−1^, underscoring the superior antibacterial effect of CDP‐based PDT. Notably, the *S*. *aureus* cells in the “CDPs + light” group revealed disrupted bacterial surfaces (Figure 5b), attributed to the in situ generation of ROS. To evaluate the antibacterial efficacy of different treatments, green‐emissive SYTO 9 and red‐emissive propidium iodide (PI) were utilized to visualize the status (live/dead) of *S*. *aureus*. As shown in Figure 5c, the PpIX‐mediated PDT (“PpIX + light” group) presented limited bacterial killing efficiency, probably due to the poor water solubility and inadequate bacteria‐targeting capacity of PpIX. In contrast, dead and aggregated *S*. *aureus* cells were observed in the “CDPs + light” group, demonstrating the superior bacterial eradication effect of CDP‐mediated PDT. Besides, the minimum bactericidal concentration (MBC) (bacterial survival ≤0.1%) of PpIX in CDPs in the “CDPs + light” group was determined to be 5 µg mL^−1^ (Figure 5d). However, the bacterial survival rate remained >70% in the “PpIX + light” group at the same concentration of PpIX (5 µg mL^−1^). The representative photographs of bacterial colonies which can directly reflect the antibacterial effects of various treatments revealed the superior therapeutic outcome of CDP‐based antibacterial PDT strategy (Figure 5e). These results demonstrated the superior bacteria‐binding ability of CDPs, which facilitated the aggregation and elimination of *S*. *aureus* during the PDT process.

**Figure 6:**
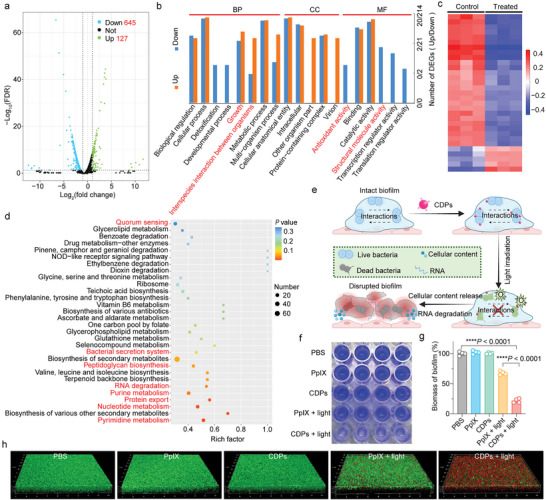
Transcriptomic analysis of bacteria after CDP‐mediated PDT treatment and evaluation of biofilm eradication. a) Volcano plot of all DEGs between the control (“PBS”) and treated (“CDPs + light”) groups. b) Histogram presenting the GO enrichment analysis results of some selected DEGs between the control and treated groups. BP: biological process, CC: cellular component, and MF: molecular function. c) Heat map of growth‐related DEGs between the control and treated groups. Red and blue colors indicate the up‐regulation and down‐regulation, respectively. d) Dot plot illustrating the KEGG enrichment analysis results of some selected DEGs between the control and treated groups. Statistical significance in (a–d) was calculated via two‐tailed Student's *t*‐test. e) Schematic illustrating the CDP‐mediated PDT for biofilm eradication. f) Photograph of the CV staining assay results of *S*. *aureus*‐based biofilms after different treatment as indicated. g) Biofilm biomass values after different treatments. The biofilm biomass was quantified by the CV absorbance of the bacterial biofilms in (f). Data are presented as mean ± SD (*n* = 4 experimental repeats). Statistical significance was calculated via one‐way ANOVA with a Tukey's post‐hoc test. ^****^ *p* < 0.0001. h) 3D confocal images of *S*. *aureus* biofilms after different treatments. Before imaging, the biofilms were stained by the live/dead bacterial viability kit.

The antibacterial mechanism underlying CDP‐mediated PDT against *S*. *aureus* was further investigated via transcriptomic analysis. More than 1.4 × 10^7^ total reads in each sample with >98% clean rate were obtained from the bacteria in the control (“PBS”) and treated (“CDPs + light”) groups. The differentially expressed genes (DEGs) between two samples were selected using the following criteria: the logarithmic value of fold change should be >2 (|log_2_(fold change)| >1) and the false discovery rate (FDR) should be less than 0.05 (FDR < 0.05). In detail, the volcano plot result revealed that a total of 772 (645 upregulated and 127 downregulated) DEGs were detected (**Figure** **6**a), and the protein–protein interaction network suggested the intricate relationships among these DEGs (Figure S17, Supporting Information). To further explore the biological alterations occurring in the bacteria in the control and treated groups, Gene Ontology (GO) and Kyoto Encyclopedia of Genes and Genomes (KEGG) enrichment analyses were performed to unravel the antibacterial mechanism of CDP‐mediated PDT. As shown in the GO enrichment analysis results (Figure 6b), altered expression profiles of the growth‐related DEGs demonstrated the suppressed bacterial viability after PDT treatment. Specifically, the heat map results unveiled 31 growth‐related DEGs, including 5 upregulated and 26 downregulated genes in the treated group (Figure 6c). Additionally, the downregulation of antioxidant activity‐related DEGs in the treated group indicated the significant antibacterial efficacy of PDT‐induced ROS generation (Figure S18a, Supporting Information), which also disrupted bacterial integrity (e.g., structural molecule activity‐related DEGs) (Figure S18b, Supporting Information). On the other hand, the KEGG pathway network revealed the complex biological changes after PDT treatment (Figure S19, Supporting Information). Noteworthy alterations were observed in bacterial cell wall/membrane‐related pathways (e.g., bacterial secretion system, peptidoglycan biosynthesis, and protein export) and intracellular metabolism‐related pathways (e.g., purine metabolism, nucleotide metabolism, and pyrimidine metabolism) (Figure 6d). Collectively, the antibacterial mechanism of CDP‐mediated PDT can be inferred as follows: The CDPs firstly bind tightly to bacterial surfaces and generate ROS in situ during light irradiation, resulting in the downregulation of structural molecule activity‐ and antioxidant activity‐related DEGs in the treated bacteria. This process damages bacterial structures, such as cell walls, thereby influencing intracellular metabolism and ultimately achieving bacterial eradication, which was demonstrated by the bacterial cell wall‐ and intracellular metabolism‐related pathways (e.g., peptidoglycan biosynthesis and nucleotide metabolism).

Microbial biofilms, as complex microbial communities, are initiated upon the recognition of solid phases by cell surface‐exposed adhesive moieties in microbes.^[^ ^41^ ^]^ Further cell–cell interactions, cell signaling, and bacterial replication foster the establishment of dense populations enveloped in a self‐produced extracellular matrix.^[^ ^42^ ^]^ The complex microenvironment and bacterial interactions within a biofilm contribute to the emergence of multi‐antibiotic resistant bacteria.^[^ ^43^ ^]^ Therefore, it is imperative to develop novel antibacterial agents capable of penetrating biofilm barriers to effectively eradicate the bacteria within the biofilms. Notably, GO and KEGG enrichment analyses revealed that the bacterial interaction‐related DEGs (e.g., interspecies interaction between organisms) (Figure 6b; Figure S18c, Supporting Information) and pathways (e.g., quorum sensing) (Figure 6d) were significantly influenced by CDP‐mediated PDT. Therefore, we employed CDP‐mediated antibacterial strategy to disrupt bacterial interactions and kill bacteria for achieving the biofilm eradication (Figure 6e). Crystal violet staining method was performed to quantify the biofilm eradication efficacy of different treatments (Figure 6f), and over 75% biomass of the biofilms was eliminated in the “CDPs + light” group (Figure 6g). Remarkably, the “CDPs + light” group effectively eradicated the biofilms by killing the bacteria, as evidenced by the highest PI signals observed among all groups (Figure 6h). Overall, CDPs efficiently penetrated the biofilms to interact with the bacteria (e.g., the bacterial surfaces), and the in situ generation of ROS disrupted bacterial interactions and further killed bacteria to eradicate biofilms.

### 2.4 CDP‐Mediated PDT for In Vivo Antibacterial Treatment and Wound Healing Promotion

**Figure 7:**
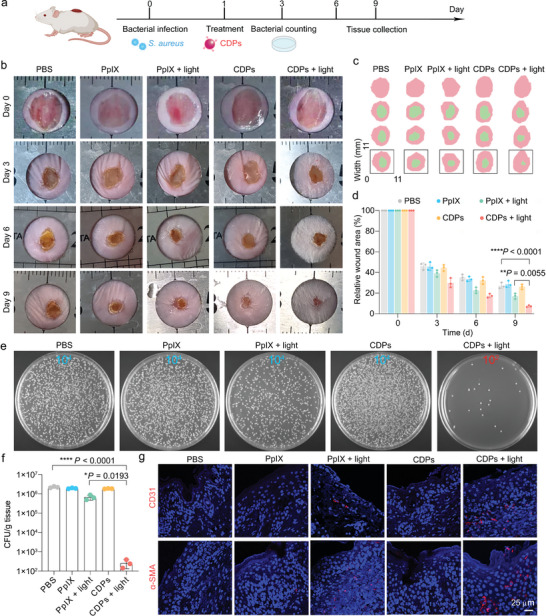
Analysis of the antibacterial and wound healing promotion effects of CDP‐mediated PDT. a) Schematic illustration of the experimental schedule. b) Representative photographs of *S*. *aureus*‐infected mice taken at different time points after various treatments as indicated. c) Overlapped simulated wound areas (the first day and the 0/3/6/9 day) within 9 days corresponding to (b). d) Histogram showing the time‐dependent relative wound areas of the mice in different groups. Data are presented as mean ± SD. *n* = 3 mice. e) Representative photographs of the bacterial colonies derived from the homogenized wound tissues at day 3. The numbers (10^2^ and 10^4^) in the images indicate the dilution factors of the original bacterial suspensions. f) Corresponding statistical histogram of the bacterial colonies in (e). Data are presented as mean ± SD. *n* = 3 mice. g) Representative immunofluorescence staining images of CD31 and α‐SMA in the wound tissue sections from the mice at day 9 after different treatments. Statistical significance in (d) and (f) was calculated via one‐way ANOVA with a Tukey's post‐hoc test. ^*^ *p* < 0.05, ^**^ *p* < 0.01, ^****^ *p* < 0.0001.

Motivated by the notable in vitro antibacterial efficacy of CDP‐mediated PDT, we further investigated the in vivo antibacterial performance of CDPs in bacteria (*S*. *aureus*)‐infected wound models (**Figure** **7**a). The representative photographs of wound regions revealed that the PpIX, “PpIX + light”, and CDPs groups presented negligible acceleration of wound healing within 9 days compared with the control (PBS) group (Figure 7b). In contrast, the infected wounds in the “CDPs + light” group exhibited a significant healing trend as time went by and almost disappeared at day 9 (Figure 7c). Furthermore, the mice in the “CDPs + light” group showed substantial hair regrowth around the wound site at day 6 (Figure 7b), indicating the remarkable promotion effect of such a treatment on wound healing.  As shown in Figure 7c, the average relative wound area in the “CDPs + light” group presented a significant reduction compared with those in the other groups, reaching 7.3% at day 9, whereas the PBS, PpIX, “PpIX + light”, and CDPs groups only decreased to 27.1%, 28.6%, 17.1%, and 26.1% at day 9, respectively (Figure 7d). At day 3, the in vivo bacterial eradication effects of different treatments were quantified through agar plate‐based bacterial colony counting of the homogenized wound tissues. Specifically, the “CDPs + light” group revealed a more than 1000‐fold reduction in the bacterial colony number compared with the other groups (Figure 7e). The average numbers of the bacterial colonies in the PBS, PpIX, “PpIX + light”, and CDPs groups were determined to be 2.3 × 10^6^, 1.9 × 10^6^, 6.7 × 10^5^, and 1.8 × 10^6^ colony forming unit (CFU)/g, respectively (Figure 7f). In contrast, the average number of the bacterial colonies in the “CDPs + light” group was only 2.6 × 10^2^ CFU/g, showing a desirable in vivo antibacterial outcome (with 99.98% bactericidal efficiency) of CDP‐mediated PDT. Next, immunostaining was performed on the slices of wound tissues to visualize the formation of new blood vessels by labeling the cluster of differentiation 31 (CD31, a marker for endothelial cells) and α‐smooth muscle actin (α‐SMA, a marker for vascular smooth muscle cells). Among all the groups, the strongest fluorescence signals of CD31 and α‐SMA were found in the “CDPs + light” group (Figure 7g), demonstrating the regeneration of abundant blood vessels attributed to the CDP‐mediated PDT.

**Figure 8:**
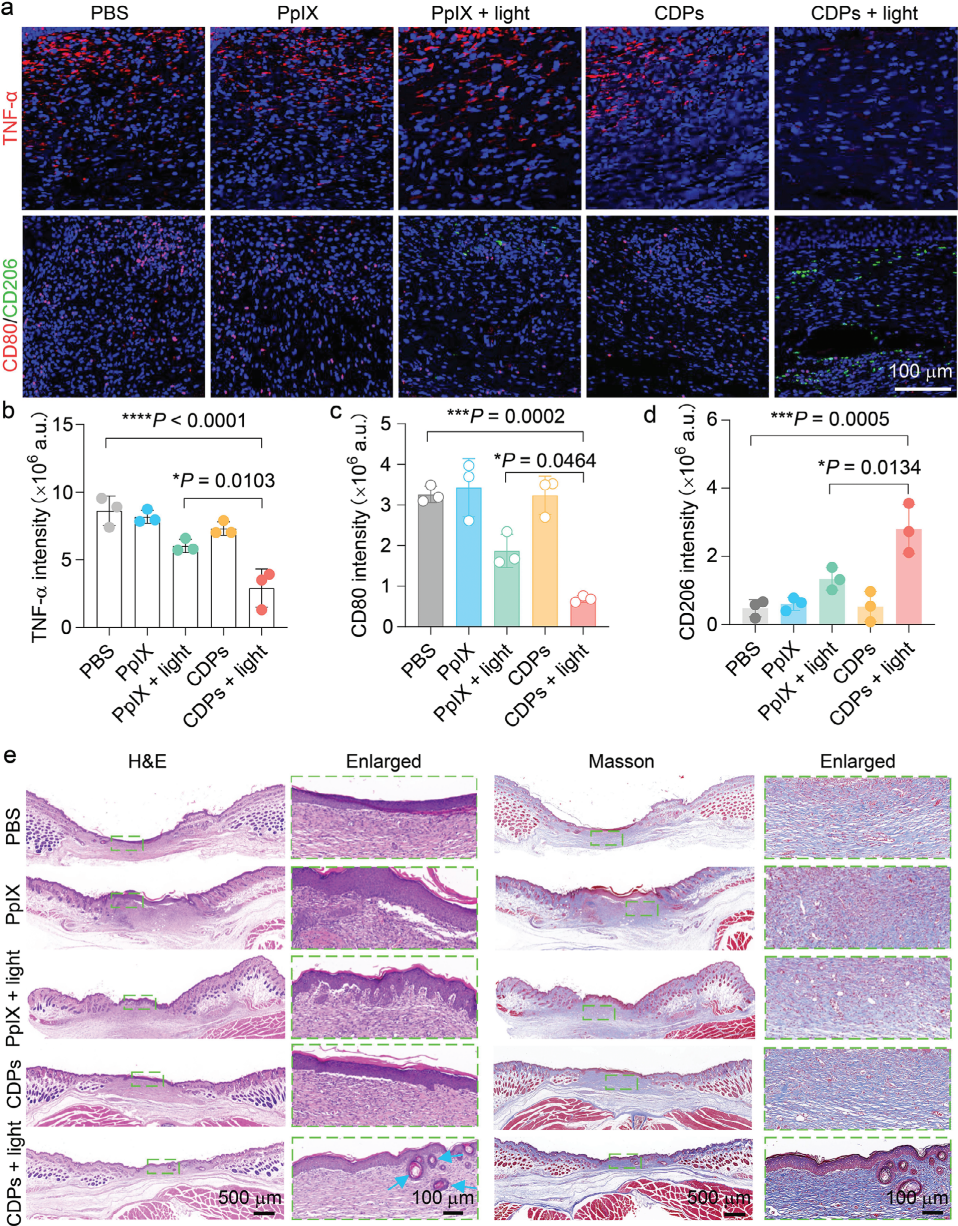
Evaluation of antiinflammation and wound healing promotion effects of CDP‐mediated PDT. a) Representative immunofluorescence images of TNF‐α and CD80/CD206 in the wound tissue sections from the mice at day 9 after different treatments. Corresponding statistical results of the fluorescence intensities for b) TNF‐α, c) CD80, and d) CD206 in (a). Data are presented as mean ± SD (*n* = 3 mice). e) Representative H&E and Masson's trichrome staining results of the wound tissue sections from the mice at day 9 after different treatments (blue arrows: blood vessels). Statistical significance in (b–d) was calculated via one‐way ANOVA with a Tukey's post‐hoc test. ^*^ *p* < 0.05, ^***^ *p* < 0.001, ^****^ *p* < 0.0001.

To comprehensively investigate the antibacterial efficacies of different treatments, we detected the levels of inflammatory indicators, including white blood cells (WBCs), tumor necrosis factor‐α (TNF‐α), and macrophages. As shown in Figure S20 (Supporting Information), the WBC level in the bacteria‐infected mice from the “PBS” group was significantly higher than that in healthy mice, suggesting the presence of bacteria‐induced inflammatory responses. In contrast, the mice in the “CDPs + light” group displayed a normal WBC level due to the effective bacterial eradication effect of the “CDPs + light” treatment. Subsequently, immunostaining was conducted to visualize the expression levels of TNF‐α, CD80 (a marker of proinflammatory M1‐type macrophages), and CD206 (a marker of antiinflammatory M2‐type macrophages). Specifically, the mice in the “CDPs + light” group exhibited the lowest TNF‐α level among all groups (**Figure** **8**a), consistent with the quantified results (Figure 8b), indicating minimal inflammatory responses in the wound sites after CDP‐mediated PDT. Additionally, the mice in the “CDPs + light” group showed a decreased level of CD80 and an increased level of CD206 compared with the other groups, confirming the high efficiency of CDP‐mediated PDT in reprogramming inflammatory environments in the infected wound regions (Figure 8a,c,d). Besides, hematoxylin and eosin (H&E) and Masson's trichrome staining were utilized to further evaluate wound regeneration in different groups. As shown in Figure 8e, the epidermis was almost regenerated in all groups, while new hair follicles, indicated by blue arrows, were almost exclusively found in the “CDPs + light” group. The Masson's trichrome staining results showed the deposition of collagen fibers in the wound regions, with the “CDPs + light” group exhibiting much denser and more organized collagen fibers compared with the other groups (Figure 8e). To evaluate the biosafety of CDPs and CDP‐mediated PDT, we collected the blood samples from the mice in the “CDPs” and “CDPs + light” groups for hemanalysis. It was revealed that CDPs and CDP‐mediated PDT had negligible influences on some blood‐related indicators (e.g., the level of RBCs) (Figure S21, Supporting Information), suggesting their good biocompatibility. Taken together, the aforementioned results demonstrate that CDP‐mediated PDT effectively eliminates the bacteria in wound regions, thereby reducing the levels of inflammatory factors in the bacteria‐infected mice and ultimately promoting the wound healing process.

## 3 Conclusion

In summary, we synthesized BCDs via a facile one‐step hydrothermal reaction, using the lixivium from natural, renewable, and cost‐effective purple rice. Subsequently, we demonstrated that the presence of WGA‐like residues endowed BCDs with a strong affinity toward G^+^ bacteria. Building on this finding, we grafted FITC molecules onto BCDs to yield CDF nanoprobe for bacterial staining. Notably, CDFs realized superselective imaging of G^+^ bacteria by labeling the bacterial cell wall, particularly the peptidoglycan component. Importantly, CDFs presented superior targeting specificity toward G^+^ bacteria even in mixed mammalian cells, G^+^ bacteria, and G^−^ bacteria, thereby enabling the detection of *S*. *aureus* (a type of G^+^ bacteria) infection in blood samples. To further exploit the potential of BCDs in biomedical applications, we conjugated BCDs with the photosensitizer PpIX to fabricate CDP nanoparticles for antibacterial PDT. Compared with free PpIX molecules, CDPs effectively bound to the cell wall of *S*. *aureus* and further induced bacterial aggregation. During white light irradiation, the intimate interactions between CDPs and bacteria amplified the killing efficiency of ROS, which disrupted the bacterial structures and interactions to realize bacterial and biofilm eradication. In the *S*. *aureus*‐infected mouse model, the CDP‐mediated PDT efficiently eliminated bacteria to modulate antiinflammatory responses, thereby promoting wound healing. Collectively, we have developed a detection probe (i.e., CDFs) and a therapeutic agent (i.e., CDPs) for G^+^ bacteria using renewable, cost‐effective, and environmentally friendly biomass by‐products. Given the facile and cost‐effective synthesis of functionalized BCDs, we believe that our strategies of CDF‐ and CDP‐based G^+^ bacteria‐targeted imaging and eradication may find potential clinical applications in the future.

## 4 Experimental Section

### Materials

Deionized water (18.2 MΩ•cm) was produced from a Milli‐Q system (Millipore, Billerica, MA). Purple rice was purchased from XIAOMIDI (Yunnan, China). FITC, urea, PpIX, lipoteichoic acid, peptidoglycan, MTT, *N*,*N*‐dimethylformamide (DMF), *N*‐hydroxysuccinimide (NHS), glycine, fluorescamine, and 1‐ethyl‐3‐(3‐dimethylaminopropyl)carbodiimide hydrochloride (EDC•HCl) were obtained from Aladdin Chemistry Co., Ltd. (Shanghai, China). DMSO, Na_2_CO_3_, NaHCO_3_, acetic acid, and acetone were ordered from Sinopharm Chemical Reagent Co., Ltd. Hoechst 33342 was purchased from Beyotime Institute Biotechnology (Shanghai, China). Live/dead bacterial viability kit, WGA‐Alexa Fluor 488, and SOSG were obtained from Invitrogen (Carlsbad, USA). DCFH‐DA was purchased from KeyGEN BioTECH Co., Ltd. Lysogeny broth (LB) was purchased from Beijing Land Bridge Technology Co., Ltd. (China). WGA ELISA kit was bought from Shanghai Enzyme‐linked Biotechnology Co., Ltd. (China). Crystal violet (CV) was purchased from Macklin Biochemical Co, Ltd (Nanjing, China). Dialysis membranes with a molecular weight cut‐off (MWCO) of 1000 and 3000 Da were purchased from Spectrum Labs (Rancho Dominguez, USA).

### Synthesis of Purple Rice Lixivium‐Derived BCDs

50 g purple rice was dispersed into 200 mL deionized water followed by bath sonication (250 W, 3 min) using an ultrasonic cleaner. Afterward, the supernatant was filtered through a filter paper to remove insoluble materials to obtain purple rice lixivium. Subsequently, 7 mL purple rice lixivium was reacted at 140 °C for 4 h using a drying oven to fabricate BCDs. Afterward, the obtained product was purified via dialysis (using the dialysis membrane with the MWCO of 1000 Da) against deionized water, which was replaced with fresh deionized water every 3 h for 4 times. Finally, the purified BCD dispersion was stored at 4 °C or lyophilized before use.

### Synthesis of CDFs

5 mg BCDs was reacted with 100 µg FITC (dissolved in DMF) in 1 mL Na_2_CO_3_/NaHCO_3_ solution (10 mM, pH = 9.5) at room temperature and stirred overnight to fabricate CDFs. Then, the above mixture was dialyzed (using the dialysis membrane with the MWCO of 1000 Da) against deionized water, which was replaced with fresh deionized water every 6 h for 4 times, to obtain purified CDF nanoprobes. Additionally, a series of free FITC solutions (solvent: DMSO) were prepared to establish a standard curve to determine the concentration of the FITC in CDFs (dissolved in DMSO) by a Duetta fluorescence and absorbance spectrometer (Horiba Scientific, USA). In this work, the concentrations of CDFs were based on BCDs.

### Synthesis of CDPs

1 mg PpIX, 1.4 mg EDC•HCl, and 2 mg NHS were mixed in 100 µL DMSO and stirred vigorously at room temperature for 4 h. Subsequently, the above mixture was reacted with 10 mg BCDs in 2 mL PBS at room temperature overnight to obtain CDP‐containing suspension. Afterward, the above suspension was dialyzed (using a dialysis membrane with the MWCO of 3000 Da) against PBS solution for 24 h to remove unreacted PpIX molecules. The purified CDP dispersion was stored at 4 °C. Additionally, a series of free PpIX solutions (solvent: DMSO) were prepared to establish a standard curve to determine the concentration of the PpIX in CDPs (dissolved in DMSO) by the Duetta fluorescence and absorbance spectrometer. In this work, the concentrations of CDPs were based on PpIX.

### Characterization of BCDs, CDFs, and CDPs

The morphology of BCDs was characterized by a transmission electron microscope (JEM‐2100, JEOL Ltd., Japan). Fluorescence, UV–vis, and 3D fluorescence spectra were collected by the Duetta fluorescence and absorbance spectrometer. The FTIR spectroscopy experiment was performed using an FTIR spectrometer (Nicolet iS50, Thermo Fisher Scientific, USA). The zeta potentials and hydrodynamic diameters of various CDs were measured by a zetasizer (Nano ZS, Malvern Instruments, UK). X‐ray photoelectron spectroscopy (XPS) analysis was conducted using an ESCALAB 250Xi system (Thermo Fisher Scientific, USA).

### Cell Culture, Bacterial Culture, and Animal Model

The murine mammary carcinoma (4T1) cell line (cat. no. KGG2224‐1) was obtained from KeyGEN BioTECH (Nanjing, China). 4T1 cells were cultured in Roswell Park Memorial Institute (RPMI) 1640 (Gibco, USA) supplemented with 10% heat‐inactivated fetal bovine serum (FBS), 100 U/mL penicillin, and 100 µg/mL streptomycin at 37 °C in a humidified atmosphere with 5% CO_2_.

Bacterial cells including *S*. *aureus*, *E*. *coli*, *P*. *aeruginosa*, *P*. *vulgaris*, and *B*. *subtilis* were cultured in LB media under shaking for 12 h at 37 °C. *M*. *luteus* bacteria were cultured in LB media under shaking for 12 h at 30 °C. To obtain the bacteria of logarithmic growth phase, the above bacteria were diluted into fresh media and cultured under shaking for 2 or 3 h. Afterward, the bacterial density was determined by recording the OD_600_ (optical density at 600 nm) values of the bacterial suspensions.

Female BALB/c mice (6–8 weeks) were purchased from Yangzhou University Medical Center (Yangzhou, China). Mice (*n* = 5/group) were housed in ventilated cage (humidity: 40–70%) with 12 h dark−light cycles at constant room temperature. All mice had access to food and water ad libitum. All the animal experiments were performed according to the relevant protocols approved by the Animal Care Committee of Southeast University (approval number: 20231204001).

### Statistical and Reproducibility Analysis

Many of the numeric data are expressed as mean ± standard deviation (SD). The significance between two groups was analyzed by two‐tailed Student's *t*‐test. For multiple comparisons, one‐way analysis of variance (ANOVA) with a Tukey's post‐hoc test was adopted. *p* values of less than 0.05 were considered significant. ^*^ *p* < 0.05, ^**^ *p* < 0.01, ^***^ *p* < 0.001, ^****^ *p* < 0.0001. “ns” stands for nonsignificant difference. All statistical analyses were performed by GraphPad Prism 9 or Excel 2019.

## Conflict of Interest

The authors declare no conflict of interest.

## Supporting information



Supporting Information

## Data Availability

The data that support the findings of this study are available from the corresponding author upon reasonable request.
