# Supplementary material for: Turning Waste into Treasure: Functionalized Biomass‐Derived Carbon Dots for Superselective Visualization and Eradication of Gram‐Positive Bacteria
Source: Adv Sci (Weinh). 2025 Jan 24;12(11):2411084. doi: 10.1002/advs.202411084 (PMC11923988; doi:10.1002/advs.202411084)
Supplement: Supplementary file 1 — Supporting Information [file ADVS-12-2411084-s001.docx]

**Turning Waste into Treasure: Functionalized Biomass-Derived Carbon Dots for Superselective Visualization and Eradication of Gram-Positive Bacteria**

*Ke-Fei Xu, Zihao Wang, Macheng Cui, Yuhan Jiang, Chengcheng Li, Zi-Xi Wang, Ling-Yi Li, Chenyang Jia, Lijie Zhang, and Fu-Gen Wu**

Dr. K.-F. Xu, Z. Wang, M. Cui, Z.-X. Wang, L.-Y. Li, C. Jia, Prof. F.-G. Wu

State Key Laboratory of Digital Medical Engineering, Jiangsu Key Laboratory for Biomaterials and Devices, School of Biological Science and Medical Engineering, Southeast University, 2 Southeast University Road, Nanjing 211189, P. R. China

E-mail: wufg@seu.edu.cn

Prof. F.-G. Wu

Department of Obstetrics and Gynecology

Zhongda Hospital

Southeast University

87 Dingjiaqiao, Nanjing, 210009, China

E-mail: wufg@seu.edu.cn

Y. Jiang

Mudi Meng Honors College, China Pharmaceutical University, Longmian Dadao Road, Nanjing 211189, P. R. China

Prof. C. Li

International Innovation Center for Forest Chemicals and Materials and Jiangsu Co-Innovation Center for Efficient Processing and Utilization of Forest Resources, Nanjing Forestry University, Nanjing 210037, P. R. China

Dr. L. Zhang

Department of Urology, Zhongda Hospital, Southeast University, Nanjing, Jiangsu 210009, P.R. China

**Additional Experiments**

**Biosafety assessment of BCDs.** 4T1 cells were seeded into 96-well plates at a density of 5 × 10^3^ cells/well and incubated for 24 h. Afterwards, the cells were treated with various concentrations (0, 30, 60, 125, 250, and 500 μg/mL) of BCDs. After incubation for another 24 h, MTT assay was performed to determine the relative cell viability.

To investigate the potential toxicity of BCDs towards bacteria, *S*. *aureus* and *E*. *coli* were selected as the representative G^+^ and G^–^ bacterial strains, respectively. The OD_600_ recording method was used to determine the bacterial growth curves after exposure to different concentrations of BCDs. In detail, the bacteria in the logarithmic growth phase were mixed with various concentrations of BCDs to obtain the treated bacterial suspensions (OD_600_ = 0.05), and the final concentrations of BCDs in the obtained suspensions were 0, 30, 60, 125, 250, and 500 μg/mL. Then, the bacterial suspension was seeded into a 96-well plate and incubated in an incubator at 37 °C and 200 rpm. The value of OD_600_ in each sample was measured by a microplate reader (Multiskan FC, Thermo Scientific, USA).

To evaluate the *in vivo* biocompatibility of BCDs, we collected the blood samples from healthy BALB/c mice for hemolysis assay. Specifically, clean red blood cells (RBCs) were obtained through removing serum from the blood samples by centrifugation (3000 rpm, 5 min). Then, RBCs were mixed with different concentrations of BCD suspensions (the final concentrations of BCDs in the obtained mixtures were 60, 120, 250, and 500 μg/mL), followed by incubation at 37 °C for 2 h. Besides, RBCs dispersed in water and PBS were set as the positive control and negative control, respectively. Finally, the absorbance of the released hemoglobin at 450 nm in each group was measured using the microplate reader to calculate the hemolysis rates.

**Bacteria-binding specificity evaluation of BCDs.** To investigate the affinity of BCDs (dispersed in PBS) towards different bacterial strains, *S*. *aureus* and *E*. *coli* (dispersed in PBS) were chosen as the representative G^+^ and G^–^ bacterial strains, respectively. Specifically, *S*. *aureus* suspension (OD_600_ = 1; solvent: PBS) and *E*. *coli* suspension (OD_600_ = 1) were set as the “PBS” groups. On the other hand, *S*. *aureus* and *E*. *coli* were separately treated with BCDs to set as the “BCDs” groups. In the “BCDs” groups, the OD_600_ value of bacterial suspensions was 1, and the final concentration of BCDs was 0.5 mg/mL. Subsequently, the above mixtures were incubated at 37 °C for 2 h without shaking. The states of bacterial suspensions were recorded by a camera at different time points (0, 0.5, 1, and 2 h) and further observed by a confocal microscope (TCS SP8, Leica, Germany).

To confirm the targeting specificity of BCDs, we incubated BCDs (dispersed in PBS) with mixed suspensions of *S*. *aureus* (dispersed in PBS) and *E*. *coli* (dispersed in PBS). Firstly, *S*. *aureus* and *E*. *coli* were stained with Hoechst 33342 (5 μg/mL) and SYTO 9 (0.5 mM) for 15 min, respectively. Afterwards, the stained bacteria were collected and dispersed in PBS for the following experiments. In detail, different concentrations (0, 0.5, 1, and 2 mg/mL) of BCDs were incubated with the mixed bacterial suspensions of Hoechst 33342-labeled *S*. *aureus* (OD_600_ = 1) and SYTO 9-labeled *E*. *coli* (OD_600_ = 1) for 2 h. Meanwhile, 0.5 mg/mL BCDs were incubated with the mixed bacterial suspension of Hoechst 33342-labeled *S*. *aureus* (OD_600_ = 1) and SYTO 9-labeled *E*. *coli* (OD_600_ = 1) for different time periods (5 min, 30 min, 1 h, and 2 h). Then, the treated bacteria were observed by the confocal microscope and analyzed by 3D imaging. A series of optical sections (*z*-series) were collected by coordinating step-by-step changes in the fine focus of the microscope with sequential image acquisition at each step. Then, the collected *z*-series were processed into a 3D representation (image) of the mixed bacteria using a Leica Application Suite X software platform.

To further validate the selective targeting capacity of BCDs towards G^+^ bacteria, we incubated BCDs with the mixture of RBCs (dispersed in PBS) and *S*. *aureus* (dispersed in PBS). In detail, *S*. *aureus* cells (OD_600_ = 1) were labeled by Hoechst 33342 (5 μg/mL) for 15 min and mixed with RBCs to obtain the mixed cellular suspensions (dispersed in PBS). Afterwards, the suspensions were divided into 2 groups, whose detailed treatments were shown as follows: (1) The suspension without further treatment (set as the “PBS” group) and (2) the suspension incubated with BCDs (0.5 mg/mL) (set as the “BCDs” group). After 1 h incubation, the suspensions were observed by the confocal microscope.

To confirm the interaction mechanism between the bacteria and BCDs, we used urea to disrupt the potential hydrogen bonding between BCDs and *S*. *aureus*. In detail, *S*. *aureus* suspensions (OD_600_ = 1; solvent: PBS) and *S*. *aureus* suspensions (OD_600_ = 1) containing 0.5 mg/mL BCDs were set as the “PBS” and “BCDs”groups, respectively. Besides, *S*. *aureus* suspensions (OD_600_ = 1) containing 5 M urea was set as the “Urea” group, and *S*. *aureus* suspensions (OD_600_ = 1) containing 0.5 mg/mL BCDs and 5 M urea was set as the “BCDs + urea” group. After 2 h incubation, the suspensions of the above groups were collected, and the bacterial counts in the suspensions were measured by flow cytometry.

**Bacteria-binding specificity and biosafety evaluations of purple rice lixivium.** To assess the potential affinity of purple rice lixivium (dispersed in PBS) towards *S*. *aureus* cells (dispersed in PBS), *S*. *aureus* cells were separately incubated with different concentrations of purple rice lixivium to obtain the treated bacterial suspensions (OD_600_ = 1), and the final concentrations of purple rice lixivium in the obtained suspensions were 0, 0.5, 1, and 2 mg/mL. Subsequently, the above mixtures were incubated at 37 °C for 2 h without shaking, and the states of bacterial suspensions were recorded by a camera.

To evaluate the toxicity of purple rice lixivium towards *S*. *aureus* cells, the bacteria in the logarithmic growth phase were mixed with various concentrations of purple rice lixivium to obtain the treated bacterial suspensions (OD_600_ = 0.05), and the final concentrations of purple rice lixivium in the obtained suspensions were 0, 30, 60, 120, 250, and 500 μg/mL. Then, the bacterial suspensions were seeded into a 96-well plate and incubated in an incubator at 37 °C and 200 rpm. The value of OD_600_ in each sample was measured by the microplate reader.

**Detection of the amino groups and WGA residues of BCDs.** According to the previous studies,^[1]^ fluorescamine was utilized to detect the primary amino groups of BCDs. Firstly, different concentrations of 100 μL glycine (dissolved in PBS) were separately mixed with 10 μL fluorescamine (dissolved in acetone) and further diluted by 890 μL PBS solutions (0.2 M, pH = 8.0), and the final concentration of fluorescamine was 0.3 mg/mL. Specifically, the final concentrations of glycine in the obtained mixtures were 25, 50, 100, 200, and 400 μg/mL. After 20 min incubation, the fluorescence spectra of the above mixtures were measured by the Duetta fluorescence and absorbance spectrometer. Therefore, a standard curve was obtained by fitting the fluorescence intensities with the corresponding concentrations of glycine at the emission wavelength of 485 nm. Afterwards, 0.5 mg/mL BCDs (dispersed in PBS) was reacted with 0.3 mg/mL fluorescamine (dissolved in acetone) in 1 mL PBS solution (0.2 M, pH = 8.0) for 20 min, and the fluorescence spectrum of the above mixture was measured by the spectrometer. Finally, the concentration of the primary amine groups in 0.5 mg/mL BCDs was determined to be similar with 73 μg/mL glycine.

The WGA residues in purple rice lixivium and BCDs were detected by using the WGA ELISA kit. According to the standard curve (Figure S7), which was measured by the WGA ELISA kit, the concentration of the WGA residues within 100 μg/mL purple rice lixivium (dispersed in PBS) and 0.5 mg/mL BCDs (dispersed in PBS) were determined to be 5.16 ng/mL and 4.08 ng/mL, respectively.

**Bacterial staining and confocal imaging.** To investigate the optimal staining conditions of CDFs (dispersed in PBS) towards bacteria, we chose *S*. *aureus* cells (dispersed in PBS) as the model G^+^ bacteria. Firstly, 50 μg/mL CDFs (FITC: 1 μg/mL) was incubated with *S*. *aureus* for different time periods (5, 10, 15, 30, and 60 min) to determine the optimal staining time. Meanwhile, the stained bacteria were observed by a confocal microscope and detected by a flow cytometer (NovoCyte 2070R, ACEA Biosciences Inc., USA). To confirm the optimal staining concentration of CDFs, we incubated *S*. *aureus* with different concentrations (50, 100, and 200 μg/mL) of CDFs for 5 min, and the stained bacteria were observed by the confocal microscope.

To explore the universal staining ability of CDFs (dispersed in PBS) towards G^+^ bacteria (dispersed in PBS) , different G^+^ (*M*. *luteus* and *B*. *subtilis*) or G^–^ (*E*. *coli*, *P*. *aeruginosa*, and *P*. *vulgaris*) bacterial strains were stained by CDFs (50 μg/mL, 5 min) and observed by the confocal microscope.

To investigate the G^+^ bacteria-binding mechanism of CDFs, we utilized *S*. *aureus*-derived lipoteichoic acid or peptidoglycan to shield the potential bacteria-binding sites of CDFs. In detail, 50 μg/mL CDFs (dispersed in PBS) were separately incubated with 1 mg/mL lipoteichoic acid and 1 mg/mL peptidoglycan at 37°C for 1 h. Afterwards, the untreated CDFs were incubated with *S*. *aureus* (dispersed in PBS) for 5 min and were set as the “CDFs” group. The lipoteichoic acid- or peptidoglycan-treated CDFs were incubated with *S*. *aureus* for 5 min and were set as the “CDFs-lipoteichoic acid” or “CDFs-peptidoglycan” group. Finally, the stained bacteria were observed by the confocal microscope and also analyzed by the flow cytometer.

**Assessment of the** G^+^ **bacteria-targeting specificity of CDFs.** To investigate the G^+^ bacteria-targeting specificity of CDFs, we incubated CDFs (dispersed in PBS) with a mixture of mammalian cells (4T1 cells) and G^+^ bacteria (*S*. *aureus*). Specifically, 4T1 cells were seeded into 96-well plates at a density of 5 × 10^3^ cells/well and incubated for 24 h. The colony forming units (CFUs) of *S*. *aureus* were determined by the flow cytometer. Afterwards, the cells were washed by PBS and mixed with *S*. *aureus* (1 × 10^8^ CFU/well) to obtain the mixed cells (dispersed in PBS). Subsequently, the mixed cells were stained by 0.5 mg/mL CDFs (FITC: 10 μg/mL) for 15 min and observed by the confocal microscope.

On the other hand, we incubated CDFs (dispersed in PBS) with a mixture of mammalian cells (4T1 cells), G^+^ bacteria (*S*. *aureus*), and G^–^ bacteria (*E*. *coli*). Similarly, 4T1 cells were seeded into 96-well plates at a density of 5 × 10^3^ cells/well and incubated for 24 h. Afterwards, the cells were washed by PBS and mixed with *S*. *aureus* (1 × 10^8^ CFU/well) and *E*. *coli* (1 × 10^8^ CFU/well) to obtain the mixed cells (dispersed in PBS). Subsequently, the mixed cells were stained by 0.5 mg/mL CDFs (FITC: 10 μg/mL) for 15 min and observed by the confocal microscope.

**Detection of bacterial infection in blood samples.** RBCs were separately mixed with different concentrations of *S*. *aureus* (1 × 10^7^ and 1 × 10^9^ CFU/mL) to yield infected blood samples. Then, 0.5 mg/mL CDFs were incubated with the above samples for 15 min, and the above cell suspensions were observed by the confocal microscope.

To demonstrate the superior G^+^ bacteria-targeting specificity of CDFs, WGA-Alexa Fluor 488 was also utilized to detect the infected blood samples. In detail, RBCs were mixed with *S*. *aureus* (1 × 10^8^ CFU/mL) to yield infected blood samples. Then, 0.5 mg/mL CDFs or 0.1 mg/mL WGA-Alexa Fluor 488 were incubated with the above samples for 15 min. Afterwards, the above suspensions were observed by the confocal microscope.

**Evaluation of singlet oxygen (^1^O_2_) generation.** To evaluate the ^1^O_2_ level in different solutions, SOSG (5 mM in methanol) was chosen as the detection probe. Specifically, 1 μL SOSG solution was mixed with 1 mL PBS, PpIX (10 μg/mL), or CDP (PpIX: 10 μg/mL) solution/suspension and the obtained mixtures were set as the “PBS”, “PpIX”, or “CDPs” groups. Besides, 1 μL SOSG solution was mixed with 1 mL PpIX solution (10 μg/mL) or CDP suspension (PpIX: 10 μg/mL), followed by continuous white light irradiation (5 mW/cm^2^, 10 min), obtaining the “PpIX + light” or “CDPs + light” group. Afterwards, the fluorescence spectra of the SOSG-treated groups were measured using the Duetta fluorescence and absorption spectrometer with an excitation wavelength of 504 nm. The characteristic peak at 518 nm of the reaction product of SOSG with ^1^O_2_ was recorded.

**Evaluation of reactive oxygen species (ROS) generation within bacteria.** Nonfluorescent DCFH-DA can be hydrolyzed by the intracellular esterases to generate 2’,7’-dichlorodihydrofluorescein (DCFH), which can be further oxidized to fluorescent 2’,7’-dichlorofluorescein (DCF) by ROS. Therefore, DCFH-DA is employed to visualize the ROS level within bacteria. Firstly, *S*. *aureus* cells were incubated with 10 μM DCFH-DA at 37°C for 20 min. Afterwards, the DCFH-DA-treated bacteria were divided into 5 groups, whose detailed treatments were shown as follows: (1) The bacteria were incubated with PBS without any other treatment (set as the “PBS” group), (2) the bacteria were treated with PpIX (5 μg/mL) for 25 min (set as the “PpIX” group), (3) the bacteria were treated with PpIX (5 μg/mL) for 15 min and then irradiated by white light (5 mW/cm^2^, 10 min) (set as the “PpIX + light” group), (4) the bacteria were treated with CDPs (PpIX: 5 μg/mL) for 25 min (set as the “CDPs” group), and (5) the bacteria were treated with CDPs (PpIX: 5 μg/mL) for 15 min and then irradiated by white light (5 mW/cm^2^, 10 min) (set as the “CDPs + light” group). Finally, the fluorescence intensities within the bacteria in each group were observed by the confocal microscope.

**Visualization of bacterial morphology after different treatments.** To investigate the potential interactions between G^+^ bacteria and PpIX/CDPs, *S*. *aureus* cells were incubated with PpIX (5 μg/mL) or CDPs (PpIX: 5 μg/mL) for 15 min and observed by the confocal microscope. To evaluate the potential damage towards bacteria after different treatments, *S*. *aureus* suspensions (OD_600_ = 0.5) were divided into 5 groups, whose detailed treatments were shown as follows: (1) The bacteria were dispersed in PBS without any treatment (set as the “PBS” group), (2) the bacteria were treated with PpIX (50 μg/mL) for 40 min (set as the “PpIX” group), (3) the bacteria were treated with PpIX (50 μg/mL) for 30 min and then irradiated by white light (5 mW/cm^2^, 10 min) (set as the “PpIX + light” group), (4) the bacteria were treated with CDPs (PpIX: 50 μg/mL) for 40 min (set as the “CDPs” group), and (5) the bacteria were treated with CDPs (PpIX: 50 μg/mL) for 30 min and then irradiated by white light (5 mW/cm^2^, 10 min) (set as the “CDPs + light” group). Subsequently, the treated bacteria were collected and washed by PBS for two times, and the obtained bacteria were fixed in 4% glutaraldehyde solution. After that, the fixed bacteria were sequentially dehydrated with 30%, 50%, 70%, 80%, 90%, 95%, and 100% ethanol solutions and observed by a scanning electron microscope (Zeiss Ultra Plus, Carl Zeiss, Germany).

**Antibacterial activity assessments.** The OD_600_ recording method was utilized to evaluate the antibacterial effects of PpIX- or CDP-mediated photodynamic therapy (PDT). In detail, *S*. *aureus* cells (OD_600_ = 0.05) in the logarithmic growth phase were mixed with different concentrations of PpIX (or CDPs) for minimum inhibitory concentration (MIC) determination. To compare with free PpIX, the concentrations of CDPs were represented by the contents of PpIX within CDPs. After 15 min incubation, the treated bacteria were irradiated by white light (5 mW/cm^2^, 10 min) and seed into a 96-well plate and incubated in an incubator (37 °C, 200 rpm). Meanwhile, the OD_600_ values in the wells were measured by the microplate reader, and the MIC_50_ values defined as the lowest concentrations of the antibacterial agents required to inhibit the growth of 50% bacteria were determined.

Live/dead staining assay and agar plate count assay were employed to further evaluate the antibacterial outcomes of different treatments. Specifically, *S*. *aureus* suspensions (OD_600_ = 0.05) were divided into 5 groups, whose detailed treatments were shown as follows: (1) The bacteria were dispersed in PBS without any other treatment (set as the “PBS” group), (2) the bacteria were treated with PpIX (5 μg/mL) for 25 min (set as the “PpIX” group), (3) the bacteria were treated with PpIX (5 μg/mL) for 15 min and then irradiated by white light (5 mW/cm^2^, 10 min) (set as the “PpIX + light” group), (4) the bacteria were treated with CDPs (PpIX: 5 μg/mL) for 25 min (set as the “CDPs” group), and (5) the bacteria were treated with CDPs (PpIX: 5 μg/mL) for 15 min and then irradiated by white light (5 mW/cm^2^, 10 min) (set as the “CDPs + light” group). To visualize the antibacterial effects in different treatments, the live/dead bacterial viability kit, which contains SYTO 9 and propidium iodide (PI), was employed to stain the treated bacteria. Briefly, SYTO 9 (green fluorescence) can label both live and dead cells when used alone, while PI can stain the damaged/dead bacteria red, reducing the SYTO 9 fluorescence when both dyes are present. To visualize the status of bacteria, the bacteria in the above groups (“PBS”, “PpIX”, “CDPs”, “PpIX + light”, and “CDPs + light”) were incubated with SYTO 9 (6 μM) and PI (30 μM) for 15 min and observed by the confocal microscope. To quantify the antibacterial efficacies of different treatments, the bacterial suspensions from the above groups (“PBS”, “PpIX”, “CDPs”, “PpIX + light”, and “CDPs + light”) were diluted 100 and 10000 times by PBS. Subsequently, 50 µL diluted bacterial suspension was poured onto an LB agar plate and cultured for 24 h at 37 °C. After that, the number of colonies on each plate was recorded.

**Investigation of CDP-mediated antibacterial mechanism.** To study the antibacterial mechanism of CDP-mediated PDT, transcriptomic analysis was utilized to explore the biological changes of *S*. *aureus* cells after treatment. Firstly, *S*. *aureus* suspensions (OD_600_ = 1) were divided into 2 groups, whose detailed treatments were shown as follows: (1) The bacteria without any further treatment (set as the “Control” group) and (2) the bacteria treated with CDPs (PpIX: 5 μg/mL) for 15 min followed by white light irradiation (5 mW/cm^2^, 10 min) (set as the “Treated” group). Afterwards, the bacteria were collected for transcriptomic analysis, and the differentially expressed genes (DEGs) between two samples were selected using the following criteria: the logarithmic value of fold change was greater than 2 (|log_2_(fold change)| > 1) and the false discovery rate (FDR) should be less than 0.05 (FDR < 0.05).

**Biofilm eradication assay.** To construct biofilms, *S*. *aureus* cells in the logarithmic growth phase were diluted using 20% LB medium. Afterwards, the diluted bacterial suspensions (OD_600_ = 0.1) were seeded into a glass-bottomed 96-well plate (200 μL/well) and incubated at 37 °C. Every 48 h, the culture medium was half-replaced with 100 µL fresh 20% LB medium. After 120 h incubation, the generated biofilms were divided into 5 groups, whose detailed treatments were shown as follows: (1) The biofilms were treated with PBS (set as the “PBS” group), (2) the biofilms were treated with PpIX (20 μg/mL) for 40 min (set as the “PpIX” group), (3) the biofilms were treated with CDPs (PpIX: 20 μg/mL) for 40 min (set as the “CDPs” group), (4) the biofilms were treated with PpIX (20 μg/mL) for 30 min and then irradiated by white light (5 mW/cm^2^, 10 min) (set as the “PpIX + light” group), and (5) the biofilms were treated with CDPs (PpIX: 20 μg/mL) for 30 min and then irradiated by white light (5 mW/cm^2^, 10 min) (set as the “CDPs + light” group). Subsequently, the CV and SYTO 9/PI staining methods were utilized to evaluate the biofilm eradication effect and viabilities of the bacterial cells within the biofilms, respectively.

For the CV staining method, the non-adherent bacteria in different groups were removed by washing with PBS. Afterwards, the biofilms were fixed by 200 μL methanol for 15 min and dried at 60 °C for 2 h. Subsequently, 100 μL CV solution (1.0 wt%) was added in each well to stain the biofilms for 5 min, and washed with PBS to remove the unbound dyes. Then, 200 μL acetic acid solutions (33.3 vol%) were separately introduced to the wells and incubated for 30 min to dissolve CV, and the absorbance of CV at 570 nm was measured using the microplate reader.

For the SYTO 9/PI staining method, the biofilms in different groups were stained by SYTO 9 (6 μM) and PI (30 μM) for 15 min and observed by the confocal microscope. Then, 3D images of the biofilms were obtained using the Leica Application Suite X software platform.

***In vivo* antibacterial activity and wound healing ability evaluations.** To construct the *S*. *aureus*-infected mouse model, BALB/c mice were firstly anesthetized by 5% isoflurane at a continuous air flow. Afterwards, an open excision wound with a diameter of 1.0 cm was cut on each back of the mice. Subsequently, 100 μL PBS solution containing *S*. *aureus* (OD_600_ = 0.5) was placed on the wound region. After 1 day, the *S*. *aureus*-infected BALB/c mice were randomly divided into 5 groups (*n* = 6/group), whose detailed treatments were shown as follows: (1) The infected wound was treated with PBS (set as the “PBS” group), (2) the infected wound was treated with PpIX (PpIX dose: 5 mg/kg) (set as the “PpIX” group), (3) the infected wound was treated with CDPs (PpIX dose: 5 mg/kg) (set as the “CDPs” group), (4) the infected wound was treated with PpIX (PpIX dose: 5 mg/kg) for 30 min and then irradiated by white light (5 mW/cm^2^, 10 min) (set as the “PpIX + light” group), and (5) the infected wound was treated with CDPs (PpIX dose: 5 mg/kg) for 30 min and then irradiated by white light (5 mW/cm^2^, 10 min) (set as the “CDPs + light” group).

For *in vivo* antibacterial activity assay, the mice in different groups (*n* = 3/group) were sacrificed at day 3, and the wound tissues were harvested. Afterwards, the obtained tissues were separately wet-weighed and homogenized in 1 mL PBS. The resulting mixtures were then diluted with saline at the dilution factors of 100 and 10000, respectively. For each sample, 50 μL of the diluted suspension was poured onto an LB agar plate and cultured at 37 ℃ overnight, and the number of colonies on each plate was recorded.

To evaluate the process of wound healing, the wound regions of all groups were photographed and recorded during the experimental period. The mice in different groups (*n* = 3/group) were sacrificed at day 9, and the blood cells were collected and analyzed using an automatic hematology analyzer (HBVET-1, Sinnowa, China). Besides, the related wound tissues were harvested for hematoxylin and eosin (H&E) staining and Masson’s trichrome staining. To investigate the formation of new blood vessels, immunofluorescence staining was used to visualize the distribution of the α-smooth muscle actin (α-SMA) and cluster of differentiation 31 (CD31). To evaluate the inflammatory levels, immunofluorescence staining was used to visualize the expression levels of tumor necrosis factor-α (TNF-α), CD80, and CD206. Besides, the fluorescence signals of TNF-α, CD80, and CD206 in different groups were quantified by the ImageJ software (version 1.52a).

**Supplementary Figures**

**
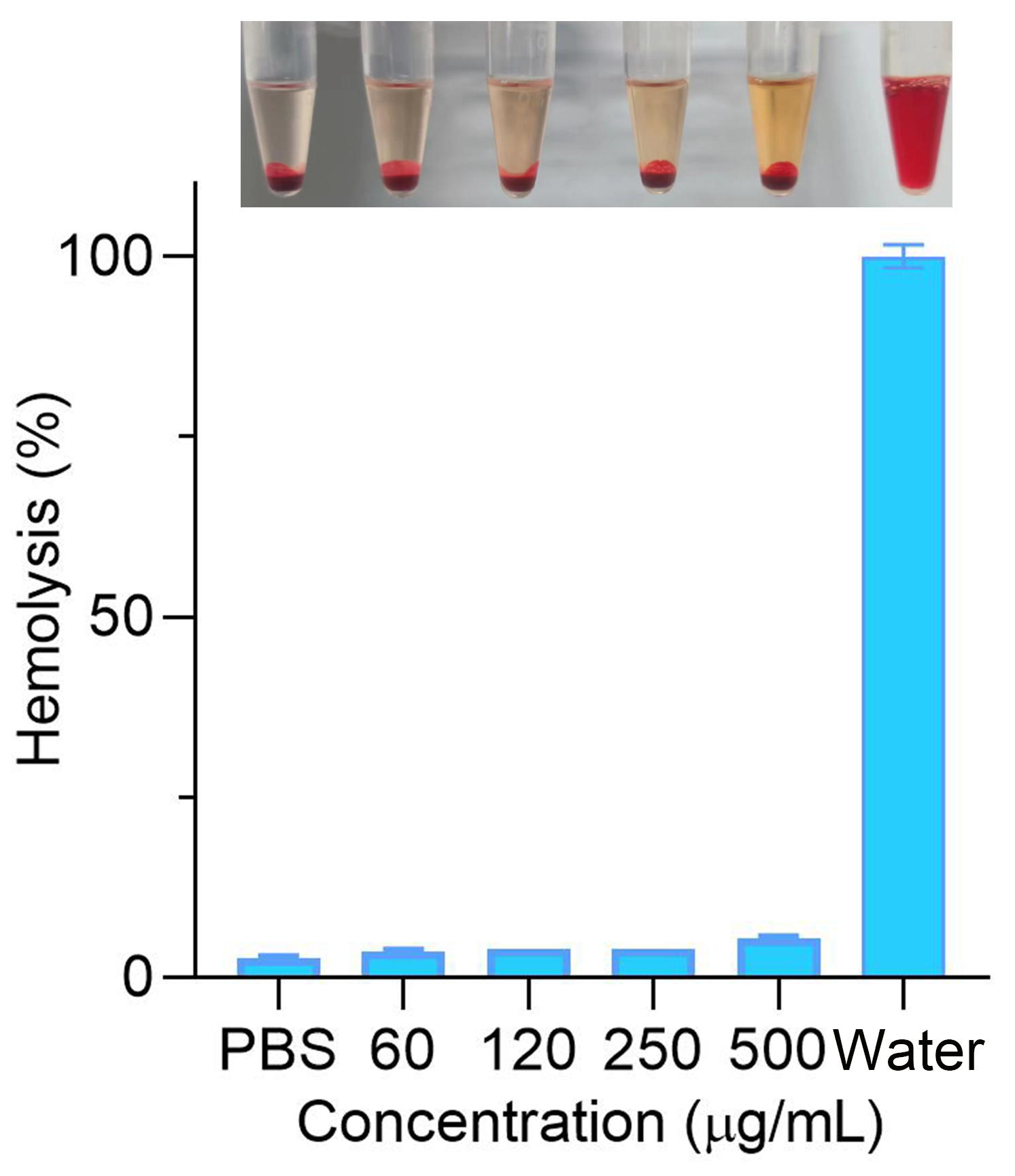
**

**Figure S1.** Hemolysis rates of RBCs after treatment with different concentrations of BCDs. RBCs dispersed in water and PBS were set as the positive control and negative control, respectively. Data are presented as mean ± SD (*n* = 3 experimental repeats).


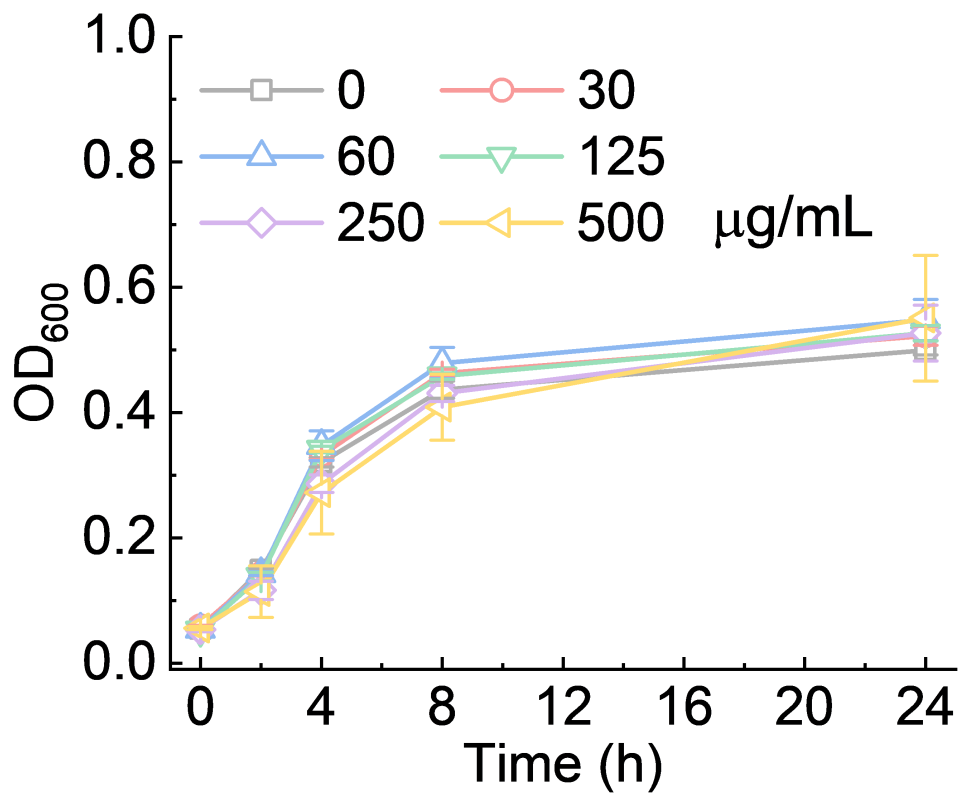


**Figure S2.** Growth curves of *E*. *coli* cells that were incubated with different concentrations of BCDs for a time period of 24 h. Data are presented as mean ± SD (*n* = 3 experimental repeats).


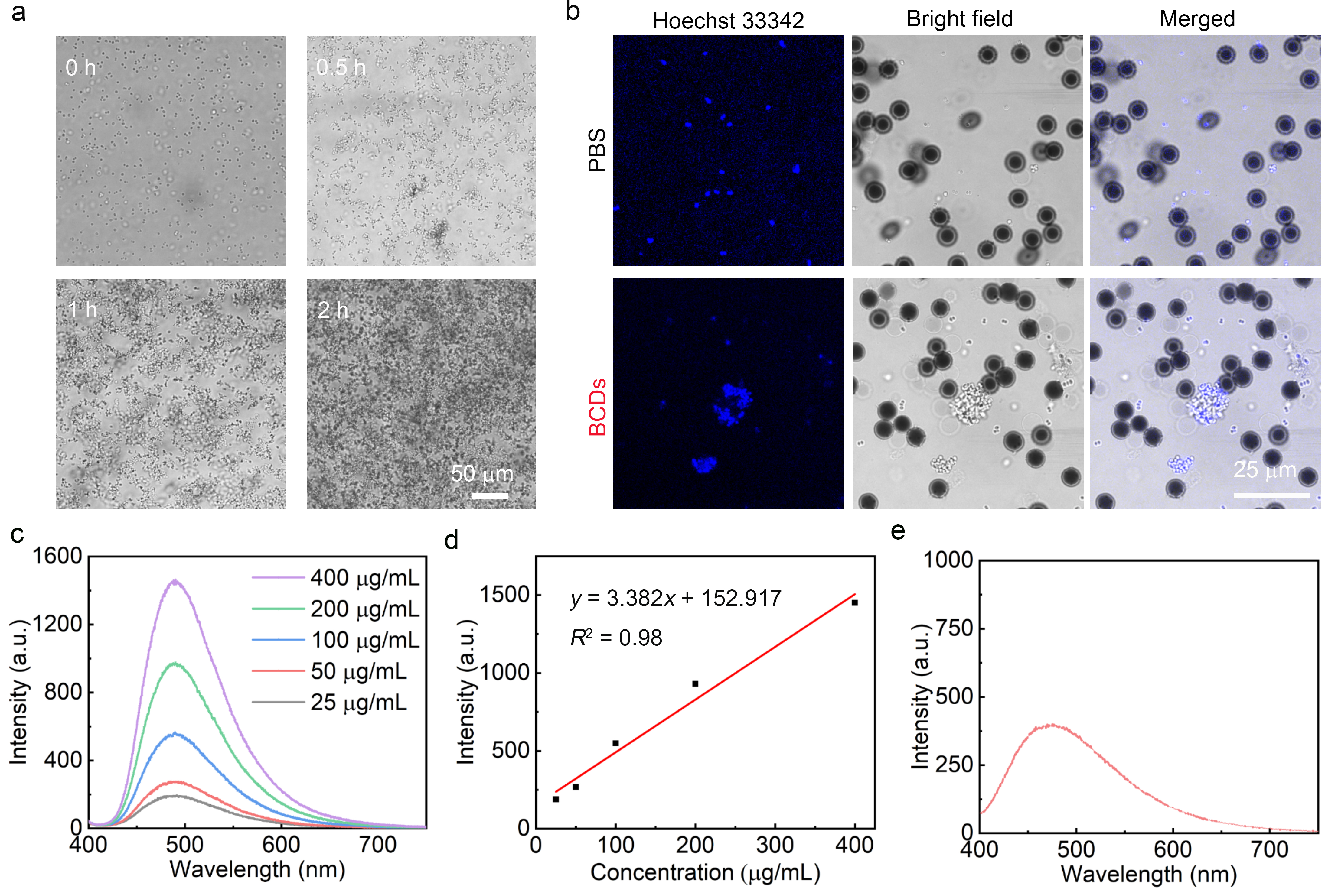


**Figure S3.** Confocal images of *S*. *aureus* cells (OD_600_ = 1) treated with BCDs (0.5 mg/mL) for different time periods.


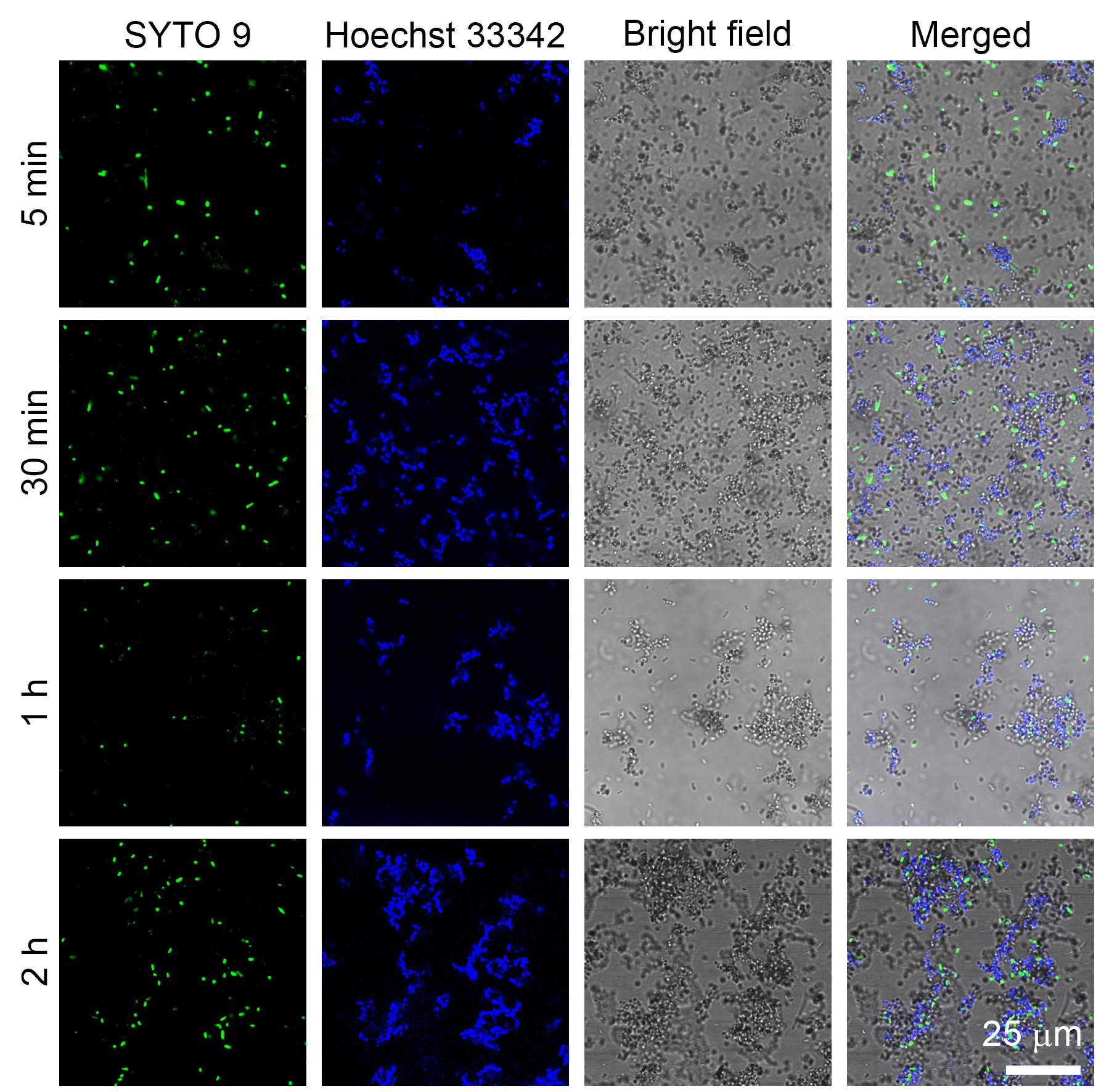


**Figure S4.** Confocal images of the mixed cells (*E*. *coli* and *S*. *aureus*) incubated with BCDs (0.5 mg/mL) for different time periods. Before imaging, the *E*. *coli* and *S*. *aureus* cells were labeled by SYTO 9 and Hoechst 33342, respectively.


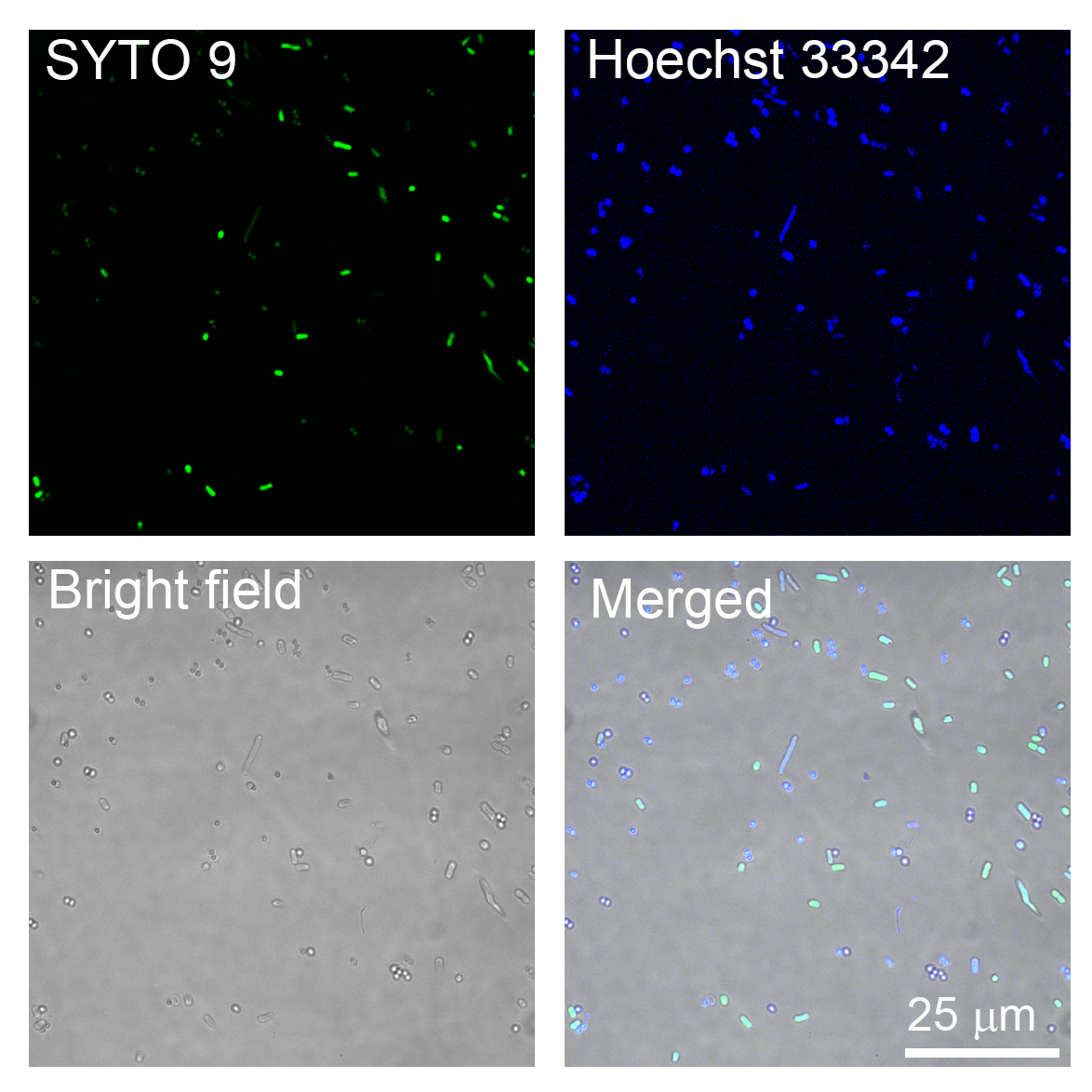


**Figure S5.** Confocal images of the mixed cells (*E*. *coli* and *S*. *aureus*) incubated in PBS for 2 h. Before imaging, the *E*. *coli* and *S*. *aureus* cells were labeled by SYTO 9 and Hoechst 33342, respectively.


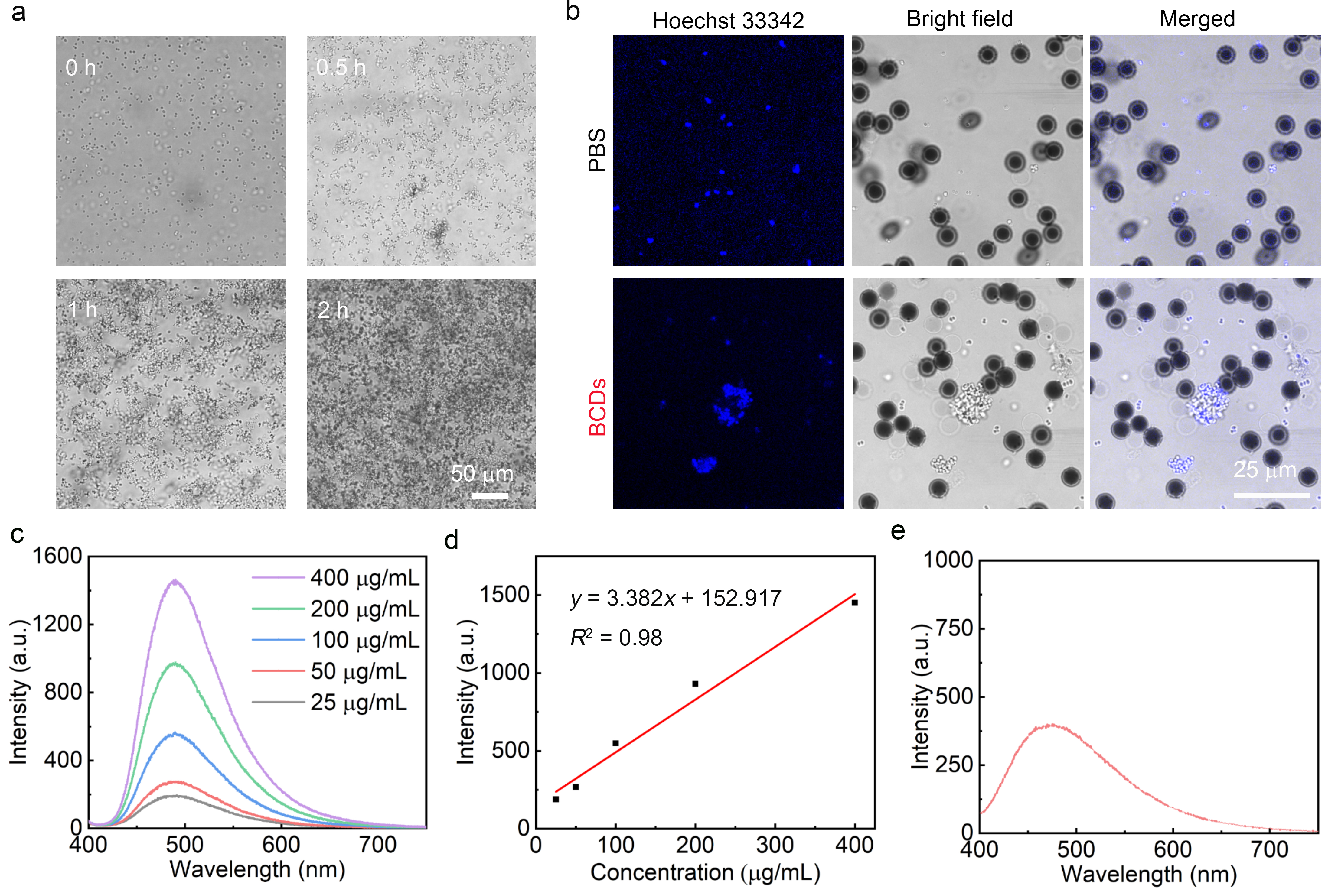


**Figure S6.** Confocal images of the mixed cells (*S*. *aureus* and RBCs) incubated with PBS or BCDs (0.5 mg/mL) for 1 h. Before imaging, the *S*. *aureus* cells were labeled by Hoechst 33342.


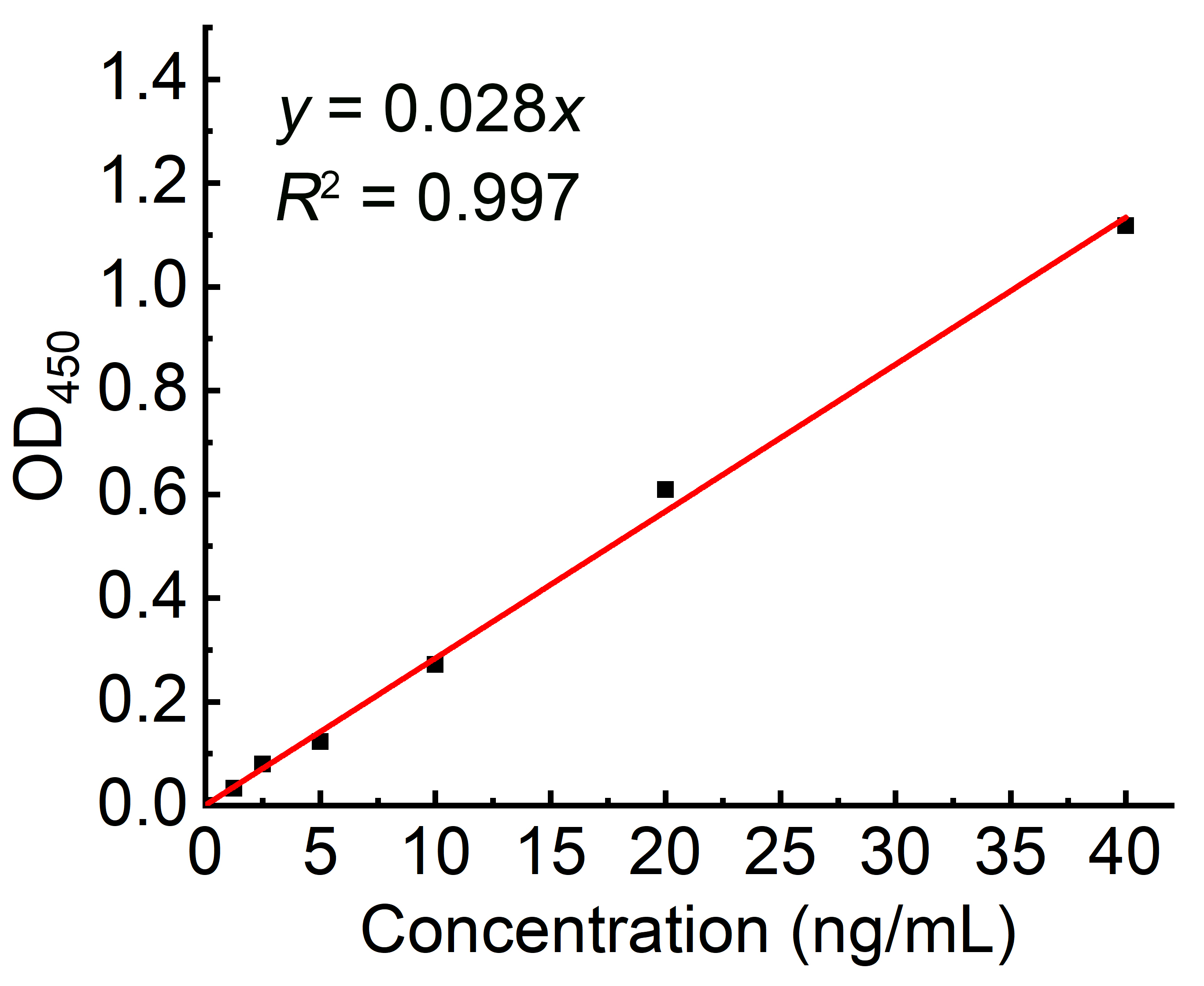


**Figure S7.** Standard curve of WGA measured by the WGA ELISA kit. The *x*-axis represents the concentrations of WGA, and the *y*-axis indicates the OD_450_ values of WGA ELISA kit-treatd standard samples measured by the Duetta fluorescence and absorbance spectrometer.


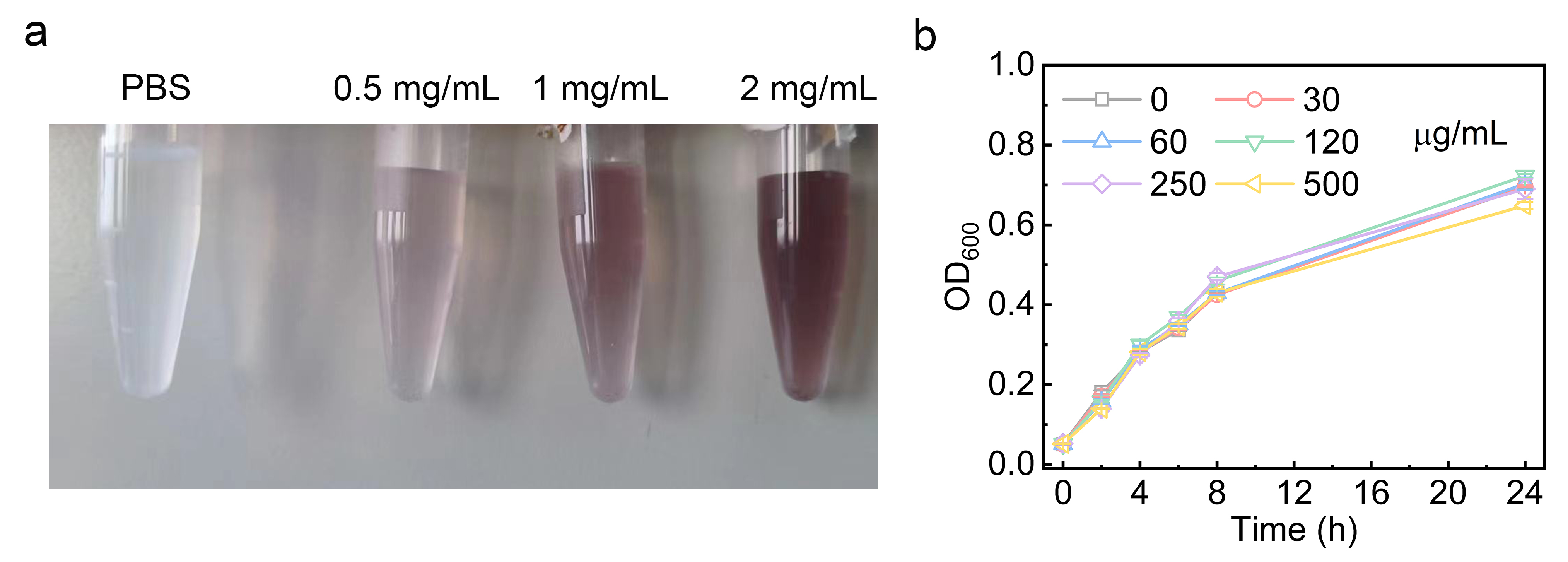


**Figure S8.** a) Photographs of *S*. *aureus* cells (OD_600_ = 1) incubated with PBS or different concentrations of purple rice lixivium for 2 h. b) Growth curves of *S*. *aureus* cells incubated with different concentrations of purple rice lixivium for a time period of 24 h. Data are presented as mean ± SD (*n* = 3 experimental repeats).


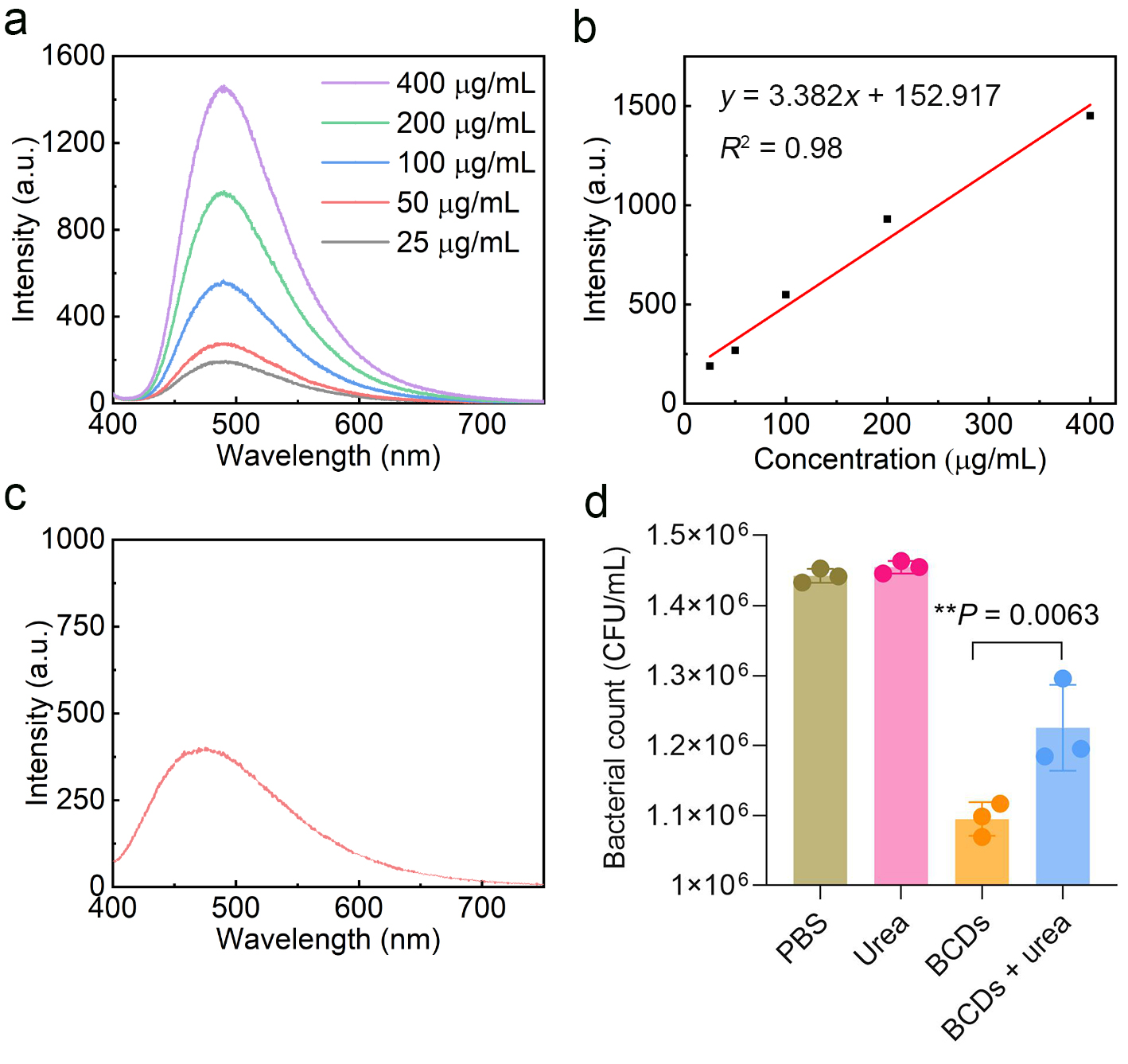


**Figure S9.** a) Fluorescence emission spectra of fluorescamine-treated glycine solutions (25, 50, 100, 200, and 400 μg/mL). Before measurement, glycine solutions were reacted with 0.3 mg/mL fluorescamine in 1 mL PBS solution (0.2 M, pH = 8.0) for 20 min. b) Fitted curve of the data in (a). c) Fluorescence emission spectrum of the fluorescamine-treated BCD suspension (0.5 mg/mL). Before measurement, the BCD suspension was reacted with 0.3 mg/mL fluorescamine in 1 mL PBS solution (0.2 M, pH = 8.0) for 20 min. d) Bacterial counts in the suspensions of different groups. Data are presented as mean ± SD. *n* = 3 experimental repeats. Statistical significance was calculated via one-way ANOVA with a Tukey’s post-hoc test. ***P* < 0.01.


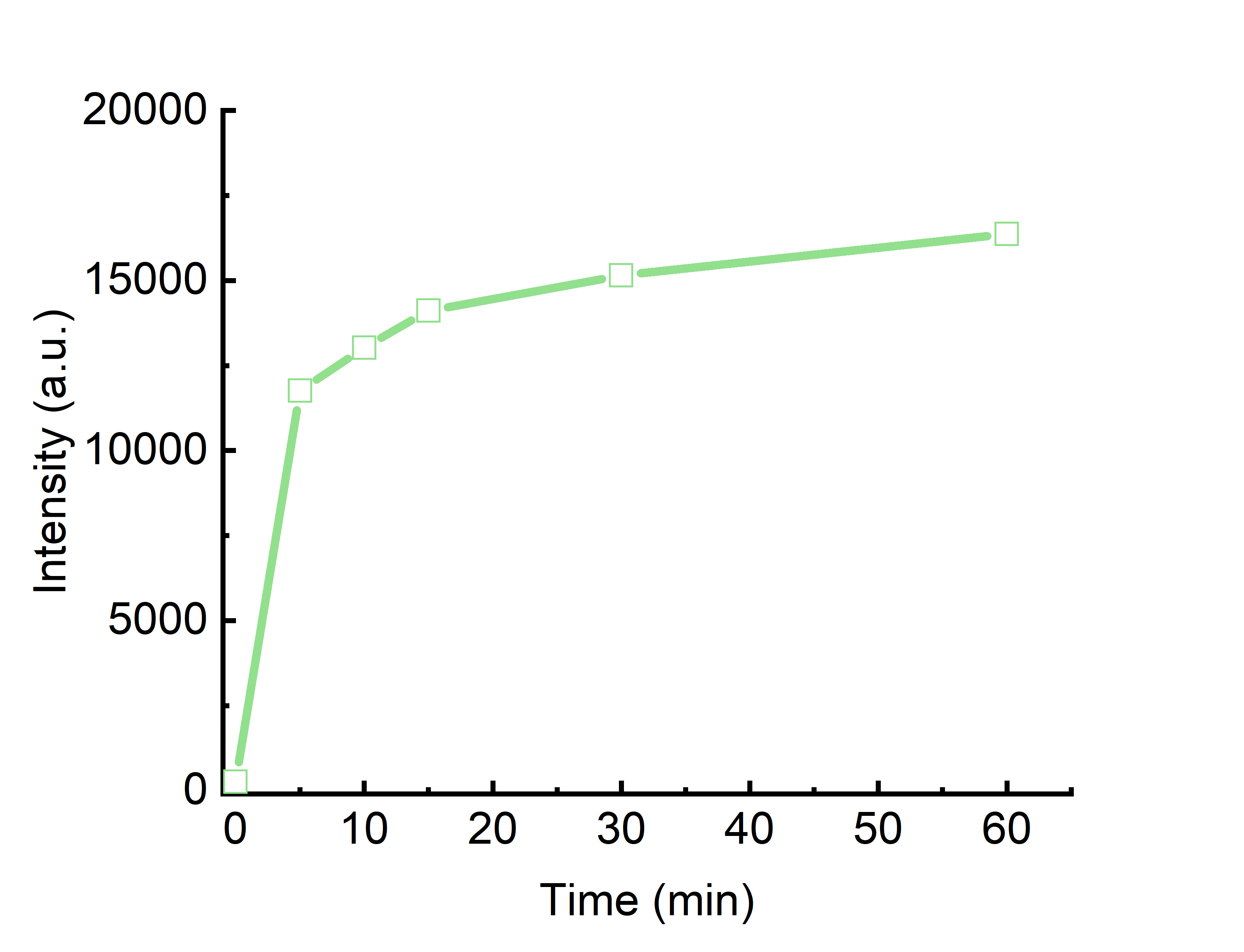


**Figure S10.** Fluorescence intensities of *S*. *aureus* cells (OD_600_ = 0.5) incubated with CDFs (50 μg/mL) for different time periods.


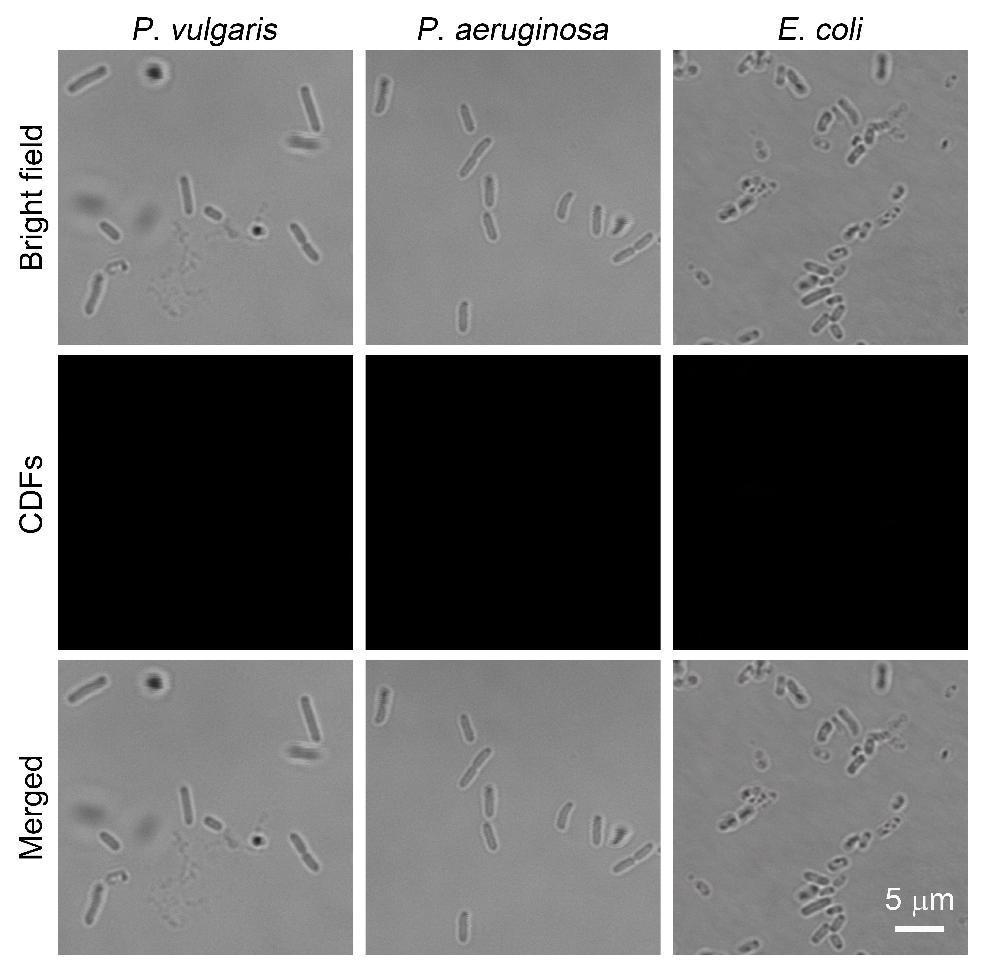


**Figure S11.** Confocal images of different G^–^ bacteria (*E*. *coli*, *P*. *aeruginosa*, or *P*. *vulgaris*) stained by CDFs (50 μg/mL) for 5 min.


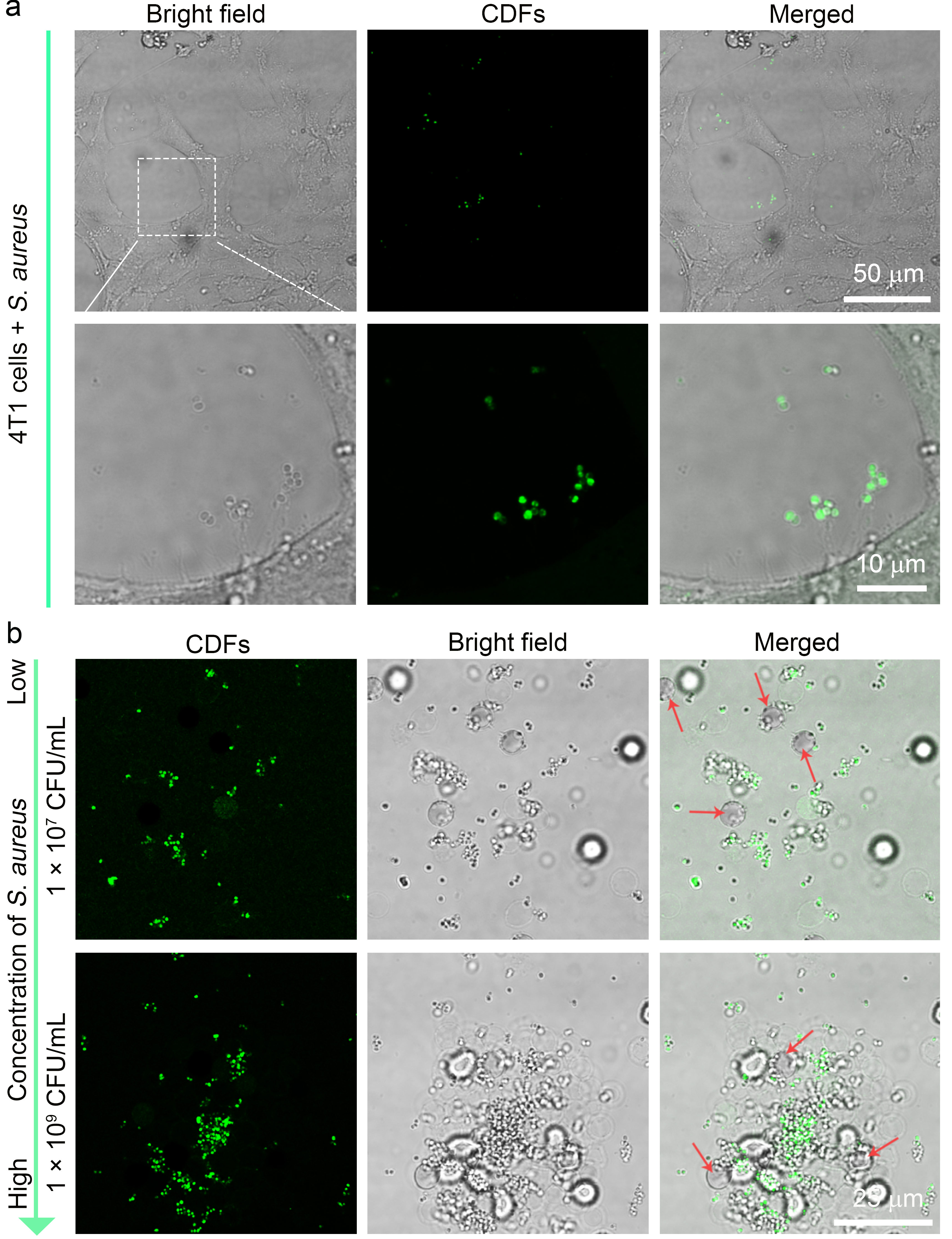


**Figure S12.** a) Confocal images of mixed cells (4T1 cells and *S*. *aureus*) stained by 0.5 mg/mL CDFs (FITC: 10 μg/mL) for 15 min. The concentration of *S*. *aureus* was 1 × 10^8^ CFU/well. b) Confocal images of the mixed cells (*S*. *aureus* and RBCs) stained by 0.5 mg/mL CDFs (FITC: 10 μg/mL) for 15 min. The red arrows indicate the unstained RBCs.


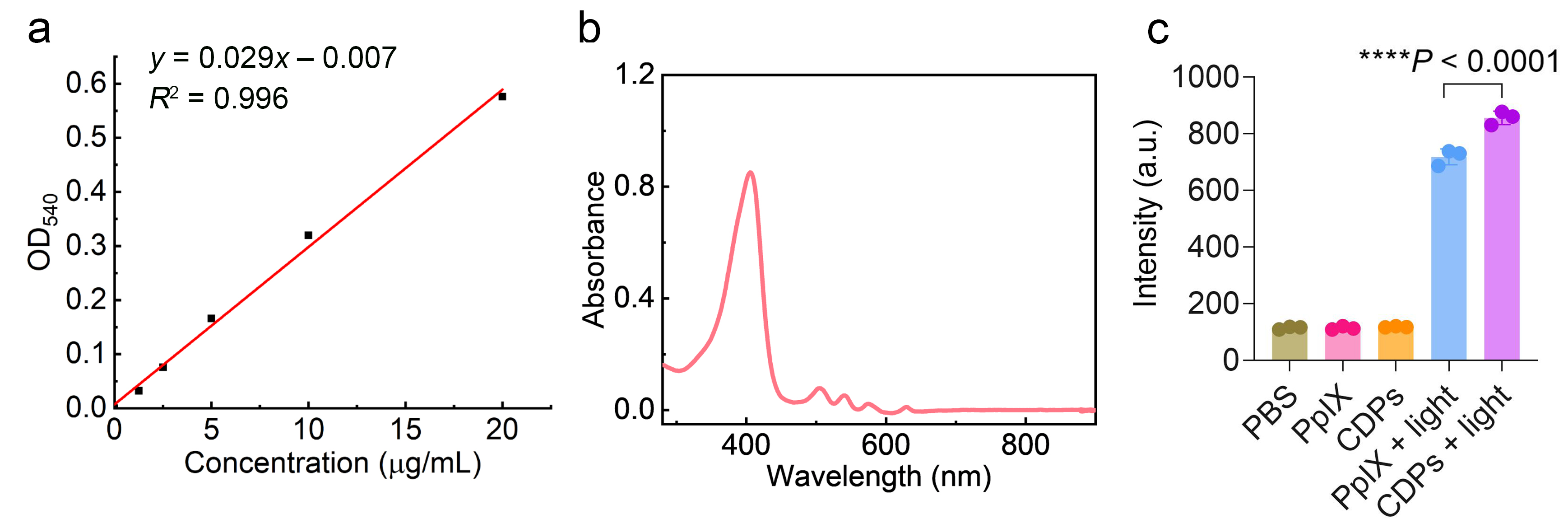


**Figure S13.** a) Standard curve of free PpIX in DMSO. The *x*-axis represents the concentrations of PpIX, and the *y*-axis indicates the OD_540_ values of PpIX measured by the Duetta fluorescence and absorbance spectrometer. b) UV–vis absorption spectrum of CDPs in DMSO. c) SOSG detection results showing the contents of ^1^O_2_ in different groups at 10 min after different treatments. Data are presented as mean ± SD (*n* = 3 mice) and analyzed by one-way ANOVA with a Tukey’s post-hoc test (*****P* < 0.0001).


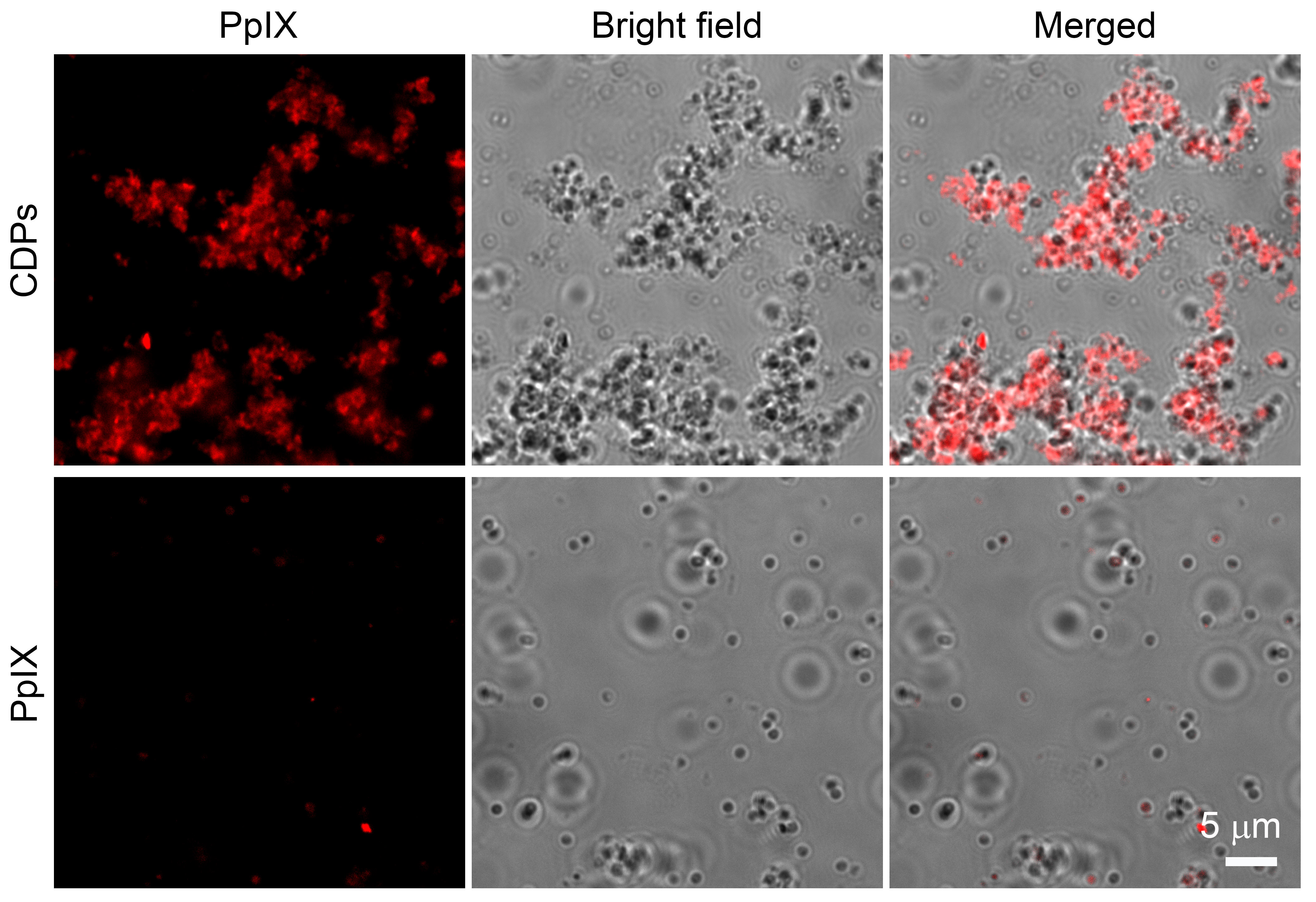


**Figure S14.** Confocal images of *S*. *aureus* cells after incubation with CDPs (PpIX: 5 μg/mL) or free PpIX (5 μg/mL) for 15 min.


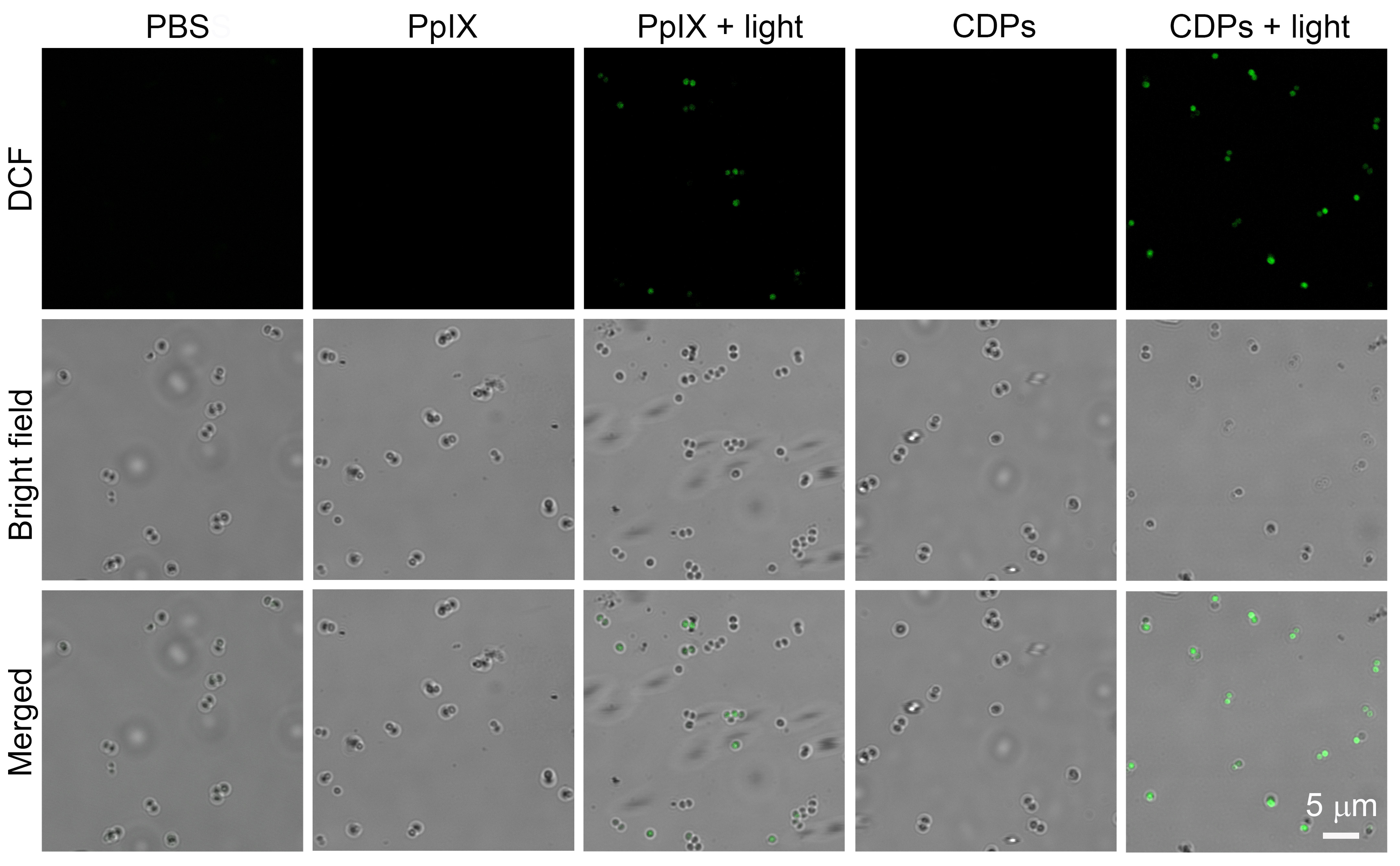


**Figure S15.** Confocal images of DCFH-DA-treated *S*. *aureus* cells in different groups as indicated.


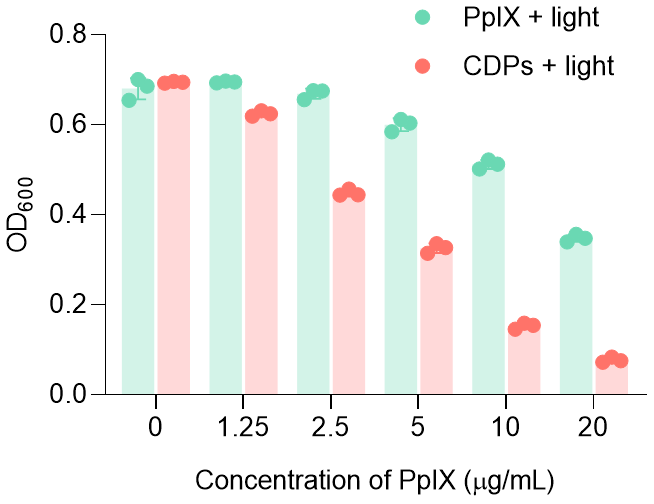


**Figure S16.** OD_600_ values of *S*. *aureus* suspensions after various treatments for 24 h as indicated. Data are presented as mean ± SD (*n* = 3 experimental repeats).


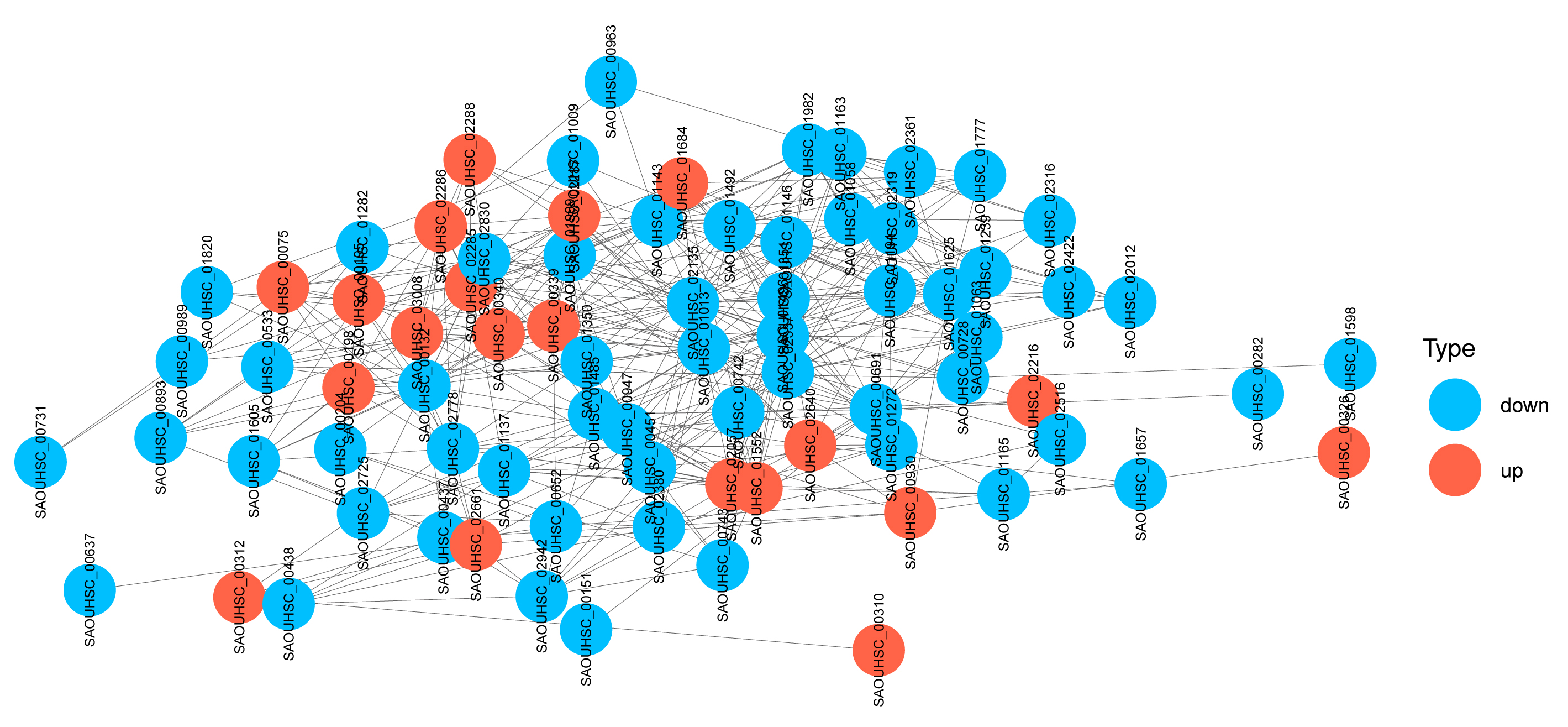


**Figure S17.** Protein–protein interaction network of the DEGs (top 200) between the control (“PBS”) and treated (“CDPs + light”) groups.


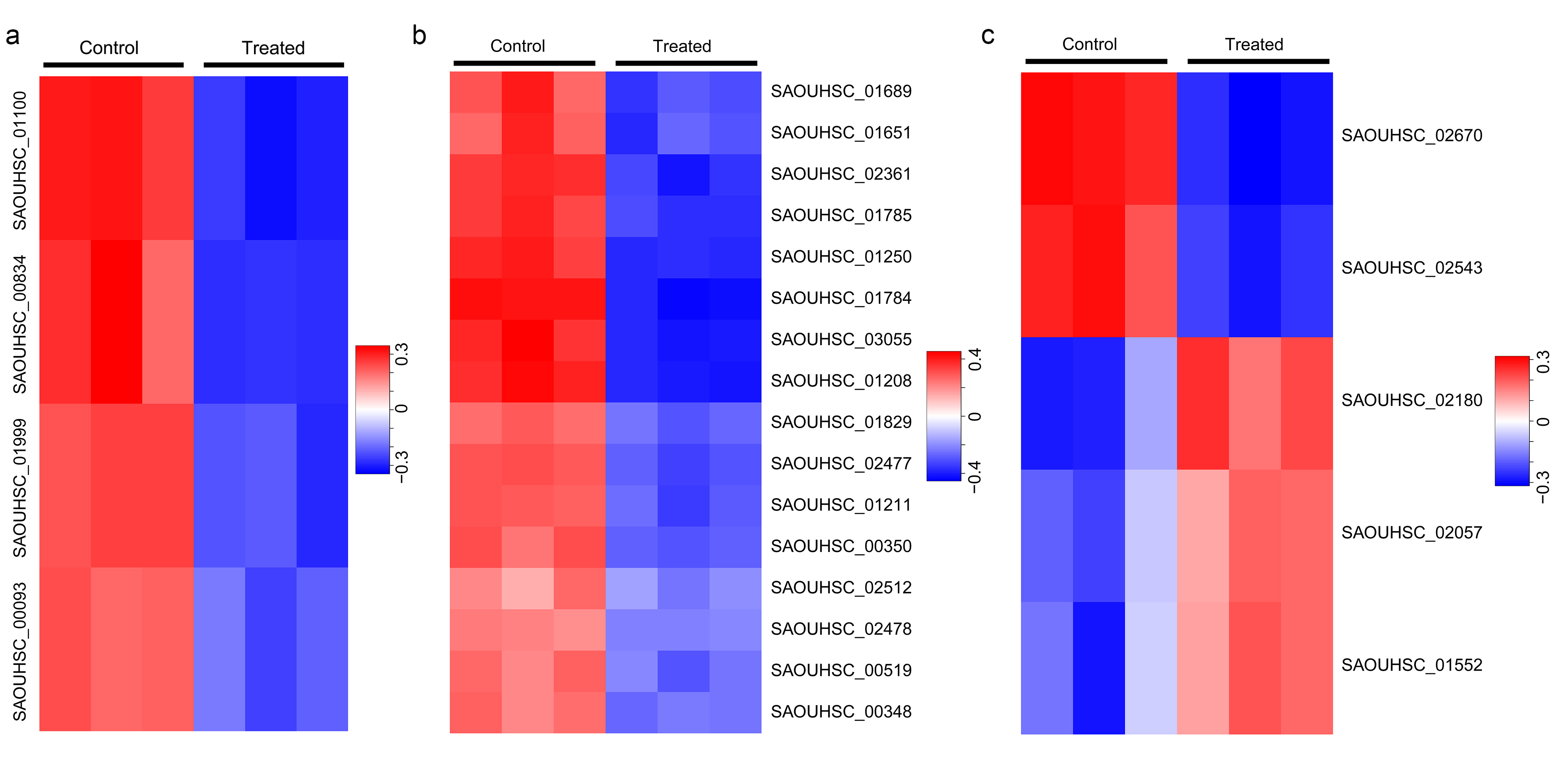


**Figure S18.** Heat map of a) antioxidant activity-related DEGs, b) structural molecule activity-related DEGs, and c) interspecies interaction between organisms-related DEGs between the control (“PBS”) and treated (“CDPs + light”) groups. Red and blue colors indicate the up-regulation and down-regulation, respectively.


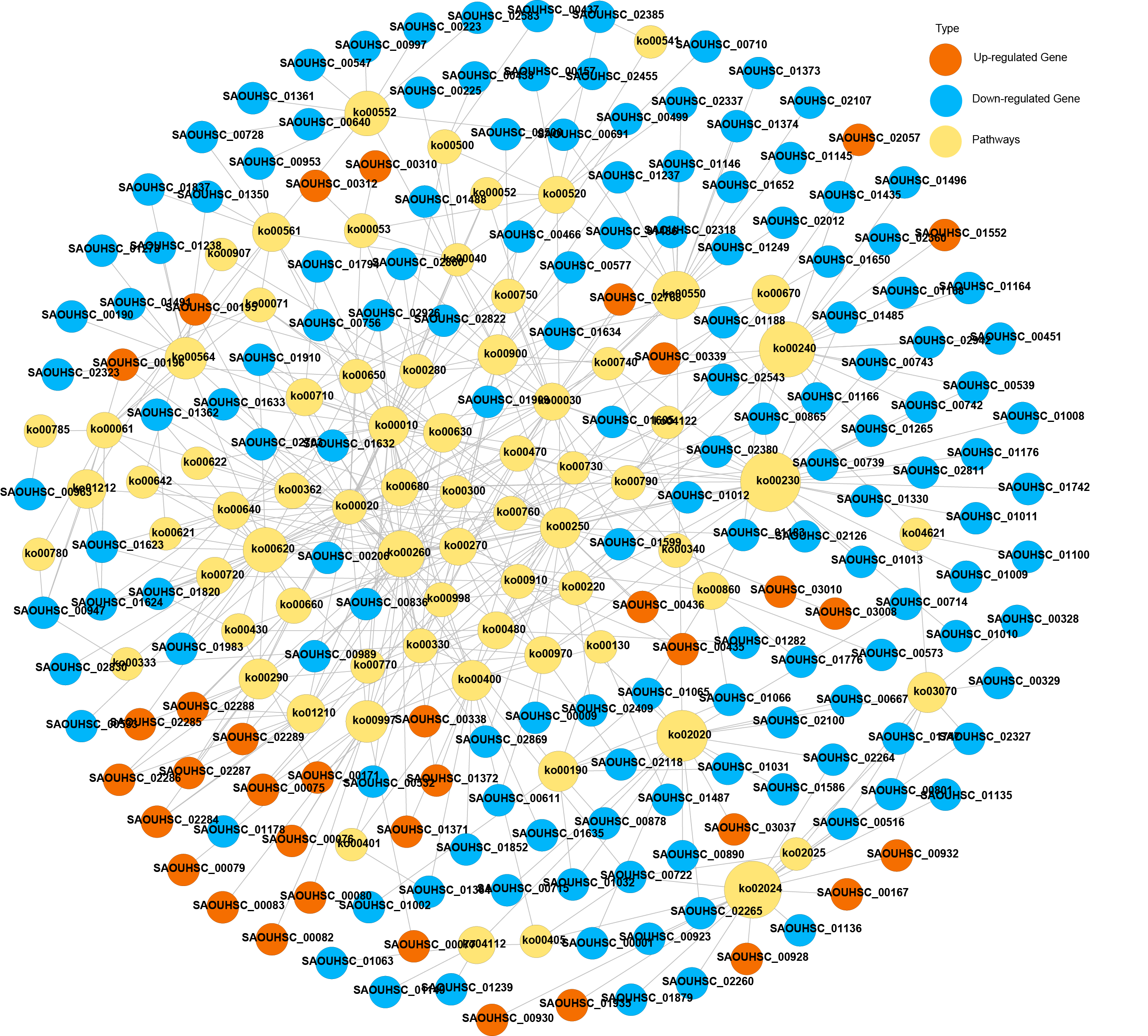


**Figure S19.** KEGG pathway network of the DEGs between the control (“PBS”) and treated (“CDPs + light”) groups.


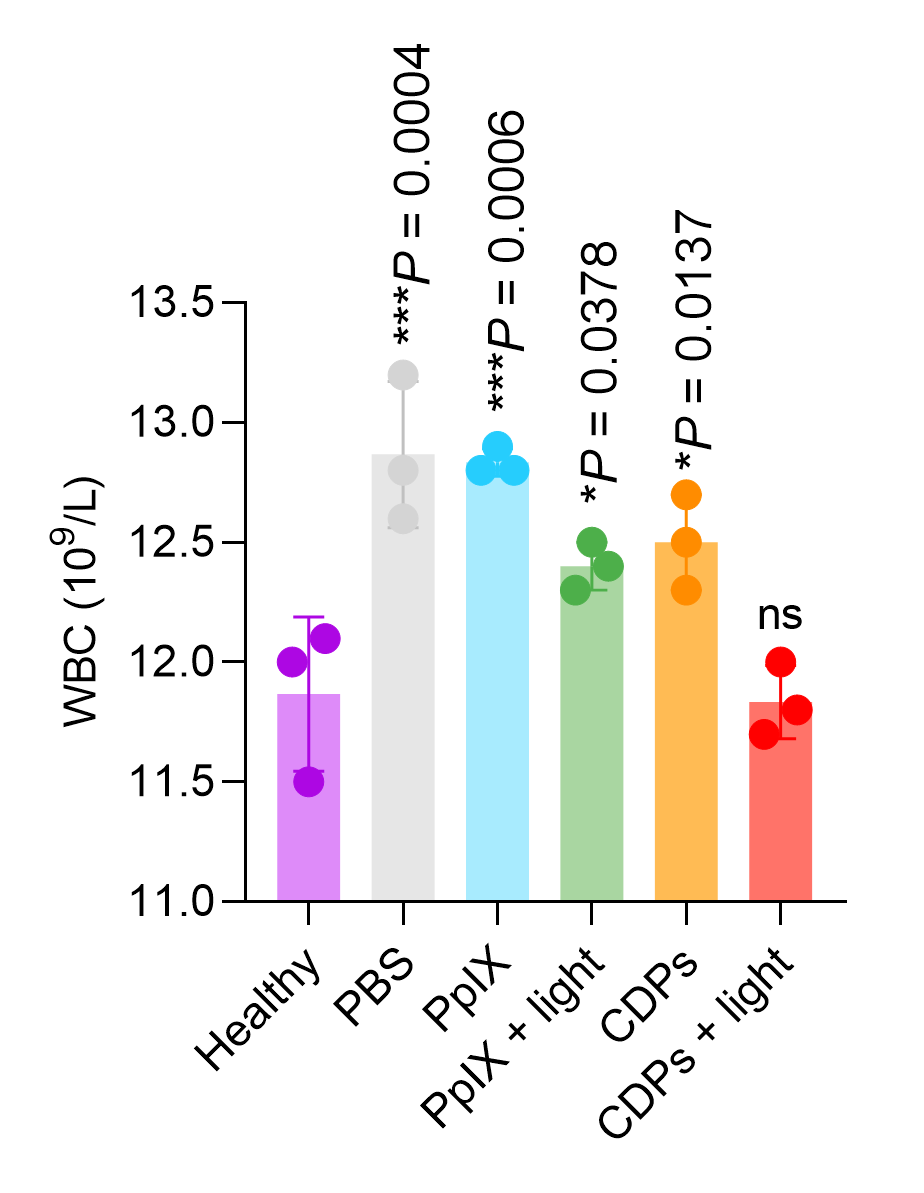


**Figure S20.** White blood cell (WBC) levels in the serums of the healthy and treated mice. Data are presented as mean ± SD (*n* = 3 mice) and analyzed by one-way ANOVA with a Tukey’s post-hoc test (**P* < 0.05, ****P* < 0.001). “ns” stands for nonsignificant difference.


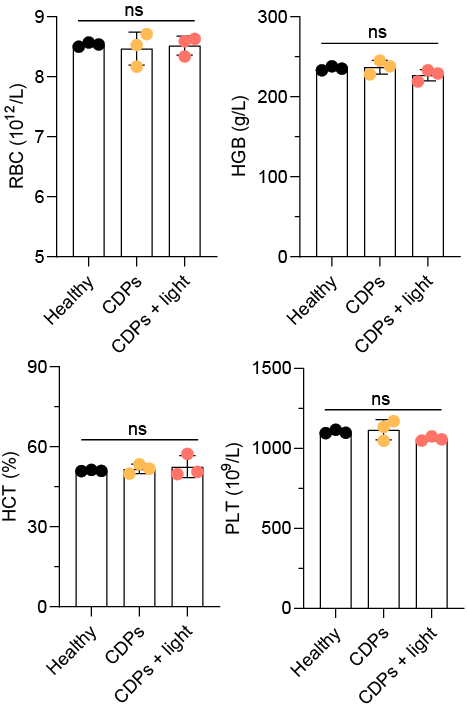


**Figure S21.** Hemanalysis results of the healthy and treated mice. Data are presented as mean ± SD (*n* = 3 mice). RBC: number of red blood cells, HGB: concentration of hemoglobin, HCT: percentage of hematocrit, PLT: number of platelets. Data are presented as mean ± SD (*n* = 3 mice) and analyzed by one-way ANOVA with a Tukey’s post-hoc test. “ns” stands for nonsignificant difference.

**References**

1. a) M. C. Miedel, J. D. Hulmes, Y. C. E. Pan, *J*. *Biochem*. *Biophys*. *Methods* **1989**, *18*, 37–52. b) R. Håkanson, L. I. Larsson, F. Sundler, *Histochemistry* **1974**, *39*, 15–23.
